# tRNA Queuosine Modification Enzyme Modulates the Growth and Microbiome Recruitment to Breast Tumors

**DOI:** 10.3390/cancers12030628

**Published:** 2020-03-09

**Authors:** Jilei Zhang, Rong Lu, Yongguo Zhang, Żaneta Matuszek, Wen Zhang, Yinglin Xia, Tao Pan, Jun Sun

**Affiliations:** 1 Division of Gastroenterology and Hepatology, Department of Medicine, University of Illinois at Chicago, Chicago, IL 60612, USA; jileiz@uic.edu (J.Z.); ronglu1@uic.edu (R.L.); yongguo@uic.edu (Y.Z.); yxia@uic.edu (Y.X.); 2 Department of Biochemistry & Molecular Biology, University of Chicago, Chicago, IL 60637, USA; zanetamatuszek@gmail.com (Ż.M.); taopan@uchicago.edu (T.P.); 3 Department of Chemistry, University of Chicago, Chicago, IL 60637, USA; article@uchicago.edu; 4 University of Illinois Cancer Center, University of Illinois at Chicago, Chicago, IL 60612, USA

**Keywords:** breast cancer, breast microbiome, gut microbiome, inflammation, micronutrient, queuosine, queuine, QTRT1, permeability, proliferation, tight junctions

## Abstract

Background: Transfer RNA (tRNA) queuosine (Q)-modifications occur specifically in 4 cellular tRNAs at the wobble anticodon position. tRNA Q-modification in human cells depends on the gut microbiome because the microbiome product queuine is required for its installation by the enzyme Q tRNA ribosyltransferase catalytic subunit 1 (QTRT1) encoded in the human genome. Queuine is a micronutrient from diet and microbiome. Although tRNA Q-modification has been studied for a long time regarding its properties in decoding and tRNA fragment generation, how QTRT1 affects tumorigenesis and the microbiome is still poorly understood. Results: We generated single clones of QTRT1-knockout breast cancer MCF7 cells using Double Nickase Plasmid. We also established a QTRT1-knockdown breast MDA-MB-231 cell line. The impacts of QTRT1 deletion or reduction on cell proliferation and migration in vitro were evaluated using cell culture, while the regulations on tumor growth in vivo were evaluated using a xenograft BALB/c nude mouse model. We found that QTRT1 deficiency in human breast cancer cells could change the functions of regulation genes, which are critical in cell proliferation, tight junction formation, and migration in human breast cancer cells in vitro and a breast tumor mouse model in vivo. We identified that several core bacteria, such as *Lachnospiraceae*, *Lactobacillus*, and *Alistipes*, were markedly changed in mice post injection with breast cancer cells. The relative abundance of bacteria in tumors induced from wildtype cells was significantly higher than those of QTRT1 deficiency cells. Conclusions: Our results demonstrate that the *QTRT1* gene and tRNA Q-modification altered cell proliferation, junctions, and microbiome in tumors and the intestine, thus playing a critical role in breast cancer development.

## 1. Introduction

Transfer RNAs (tRNAs) read the genetic code and are essential for the proliferation, fitness, and adaptation of the cells in the body. Emerging roles of tRNA in various human diseases have been implicated through genetic mutations, post-transcriptional modifications, overexpression, and RNA–protein interactions. tRNA-derived small RNA fragments (tRFs) have been shown to be associated with breast cancer in the extracellular vehicles and blood, indicating that tRFs may be useful as biomarkers for breast cancer progression and treatment [1,2,3,4]. Both tRNA and tRFs function in cells, and in turn their effect on breast cancer tumor development and progression may be modulated by microbiome-dependent queuosine (Q)-tRNA modifications. tRNA Q-modification has been associated with various forms of tumors [5]. For example, the severity of liver tumorigenesis was found to be inversely proportional to the amount of Q-modifications in total cellular tRNAs. On the other hand, other tumor types can show opposite trends. In particular, the potential role of the microbiome/Q through tRNA modification has not been explored in breast cancer, although the lack of salvage capabilities following turnover of Q-modified tRNA was said to account for the effect of 50%–60% Q-deficiency in the MCF7 breast adenocarcinoma cells [6].

Queuosine is the nucleoside with a modified base designated as queuine, which is an evolutionary ancient compound. The bacteria are unique in their ability to synthesize queuine and pass it on to plants and animals. For eukaryotes, queuine, recovered from digested food or gut microbiota, is converted to queuosine by placing it in the wobble position of several rRNAs, including aspartic acid, asparagine, histidine, and tyrosine [7]. Queuine is transported into the cell through a rapidly saturating low *K*_m_ component and a slower-uptake high *K*_m_ component, and both cytosol and mitochondrial rRNA are modified by the queuine-insertase enzyme. Inside the cells, RNA polymerase III is used for transcription of cytosolic tRNA in the nucleus, while mitochondrial-directed RNA polymerase is used for the transcription of mitochondrial tRNA in mitochondria. Queuine base is incorporated into cytosolic and mitochondrial tRNAs on the mitochondrial membrane where they co-localize with the queuine-insertase complex, which includes QTRT1 and queuine-tRNA ribosyltransferase domain containing 1 (QTRT2 or QTRTD1) subunits [5]. In humans, queuine produced by the gut microbiome is the substrate of the heterodimeric enzyme of QTRT1/QTRT2 for tRNA modification, and QTRT1 is the catalytic subunit that is conserved from bacteria to humans [8]. Thus, Q modification in human cells depends on the intestinal microbiome. tRNA Q-modifications occur specifically in four cellular tRNAs at the wobble anticodon position that is directly involved in decoding genetic information [9,10]. tRNA Q-modifications also affect the cellular small RNA pools that can modulate cell physiology through RNA–protein interactions [8].

Queuine is a microbial-derived micronutrient that influences translational fidelity and ultimately the evolutionary fate of the genome [5]. Q-modification of rRNA could affect translation at a number of stages, such as the efficiency of tRNA aminoacylation in the presence of queuosine. Studies in eukaryotes indicate that effects of Q-modification on the codon–anticodon recognition may be subtle, and no essential difference was observed in the rate or the extent of protein synthesis between Q-containing and Q-lacking rRNA. Q-modification is limited by the availability of queuine.

In this study, we aim to (1) examine microbiome-dependent Q-tRNA modification and its impact on breast tumor growth and gene expression, and (2) to identify the mechanisms of tRNA Q-modification-dependent cellular phenotypes in vitro and in vivo. We generated single clones of complete QTRT1-KO in human breast cancer MCF7 cells. To confirm the role of QTRT1 in breast cancer cells, we knockdown QTRT1 in the MDA-MB-231 breast cancer cells. These clones were used for cellular experiments in vitro and mouse model studies in vivo. We identified the functions of the QTRT1 gene in regulating critical genes in cell proliferation, tight junction, and migration with human breast cancer cells and a breast tumor mouse model. Importantly, we find evidence that the microbiome is involved in breast cancer development in vivo. Our results demonstrate that at the cellular and molecular levels, altered tRNA Q-modifications and dysbiosis can play critical roles in breast cancer.

## 2. Results

### 2.1. Establishment of QTRT1 Knockout (KO) Clone in Breast Cancer Cell Line

**Figure 1:**
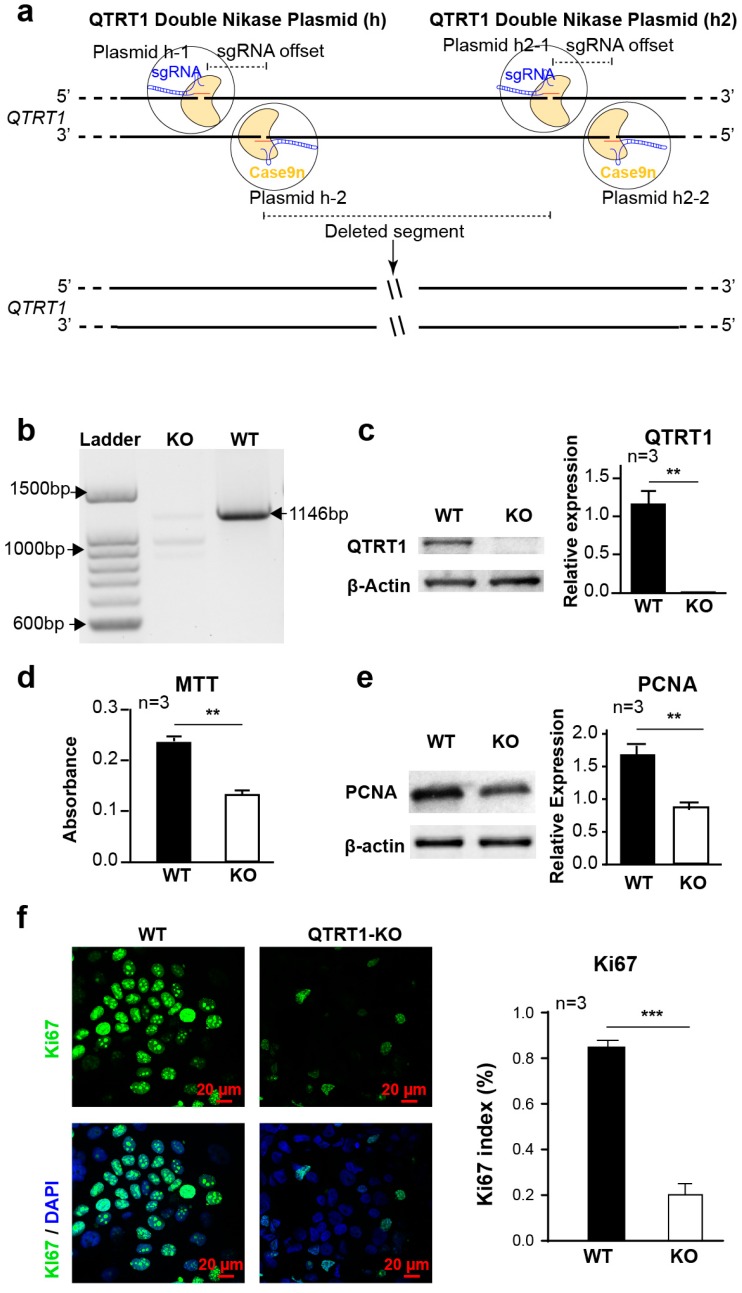
Knockout of Q tRNA ribosyltransferase catalytic subunit 1 (QTRT1) suppressed MCF7 breast cancer cell proliferation. (**a**) Schematic illustration of double-stranded DNA breaks using a pair of Cas9 D10A nickases (Cas9n). (**b**) The deletion of QTRT1 in MCF7 cells was confirmed using PCR with primers specific for the *QTRT1* gene. (**c**) Western blot analysis of wildtype (WT), QTRT1-knockout (KO) MCF7 cells generated using Double Nickase Plasmids after treating for 72 h. Mean ± SD, *n* = 3; ** *p*-value < 0.01, two-tailed Welch’s *t*-test. (**d**) MTT assay to show cell proliferation of WT and QTRT1-KO MCF7 conducted at 48 h after seeding the same number of cells. Mean ± SD, *n* = 3; ** *p*-value < 0.01, two-tailed Welch’s *t*-test. (**e**,**f**) Cell proliferation markers of PCNA (proliferating cell nuclear antigen) and Ki67 in WT and QTRT1-KO MCF7 cells were detected using Western blot (E) and immunofluorescence staining (F), respectively. Immunofluorescence staining of Ki67 and DAPI were performed in the cells, and Ki67 index (Ki67 stained cells/total cells) was calculated. Mean ± SD, *n* = 3; ** *p*-value < 0.01, *** *p*-value < 0.001, two-tailed Welch’s *t*-test.

To investigate the effects of QTRT1 on breast cancer tumor growth, we used the Double Nickase Plasmid to suppress the expression of QTRT1 in MCF7 cells (Figure 1a). After plasmid transfection and puromycin selection, the DNA and protein expression levels of QTRT1 were below the threshold of detection in MCF7 breast cancer cells (Figure 1b,c). QTRT1-special amplicon upon PCR amplification of QTRT1-KO cells was absent (Figure 1b), which was further confirmed by genomic sequencing of the PCR products (Figure S1a). The reduced QTRT1 expression in the knockout clone was persistent in different passages of cells, indicating that we established stable QTRT1-KO in human MCF7 cells. To confirm the role of QTRT1 in breast cancer cells, we further knockdown QTRT1 in the MDA-MB-231 breast cancer cells (Figure S2a,b). This model will help us to understand whether a knockdown of QTRT1 is sufficient to induce all of these phenotypes.

We measured the Q-modification levels of the MCF7 wildtype (WT) and QTRT1-KO cells using the standard APB gel method (Figure S1b). We found that the WT cells had nearly stoichiometric levels of Q-modification, whereas our QTRT1-KO cells showed significantly reduced Q-modification levels (~25%). This incomplete elimination of Q-modification may be derived from either incomplete removal of potential QTRT1 splice isoforms in our cells, or the presence of an unknown gene or pseudogene that can also Q-modify tRNA. In any case, our KO cells did show a significant reduction of Q-levels, consistent with our experimental design.

### 2.2. Knockout of QTRT1 Suppressed Cell Proliferation and Migration

After establishing the QTRT1-KO single clone in MCF7 cells, we examined cell proliferation by the MTT assay, which assesses viable cell metabolic activity. We found that cell proliferation was significantly reduced in QTRT1-KO MCF7 cells (*p* < 0.01) (Figure 1d) compared with its parental cells. The suppressed proliferation ability in the cells was further verified by significantly decreasing the expression of PCNA and Ki67, the markers for cell proliferation, in QTRT1-KO MCF7 cells (Figure 1e,f).

**Figure 2:**
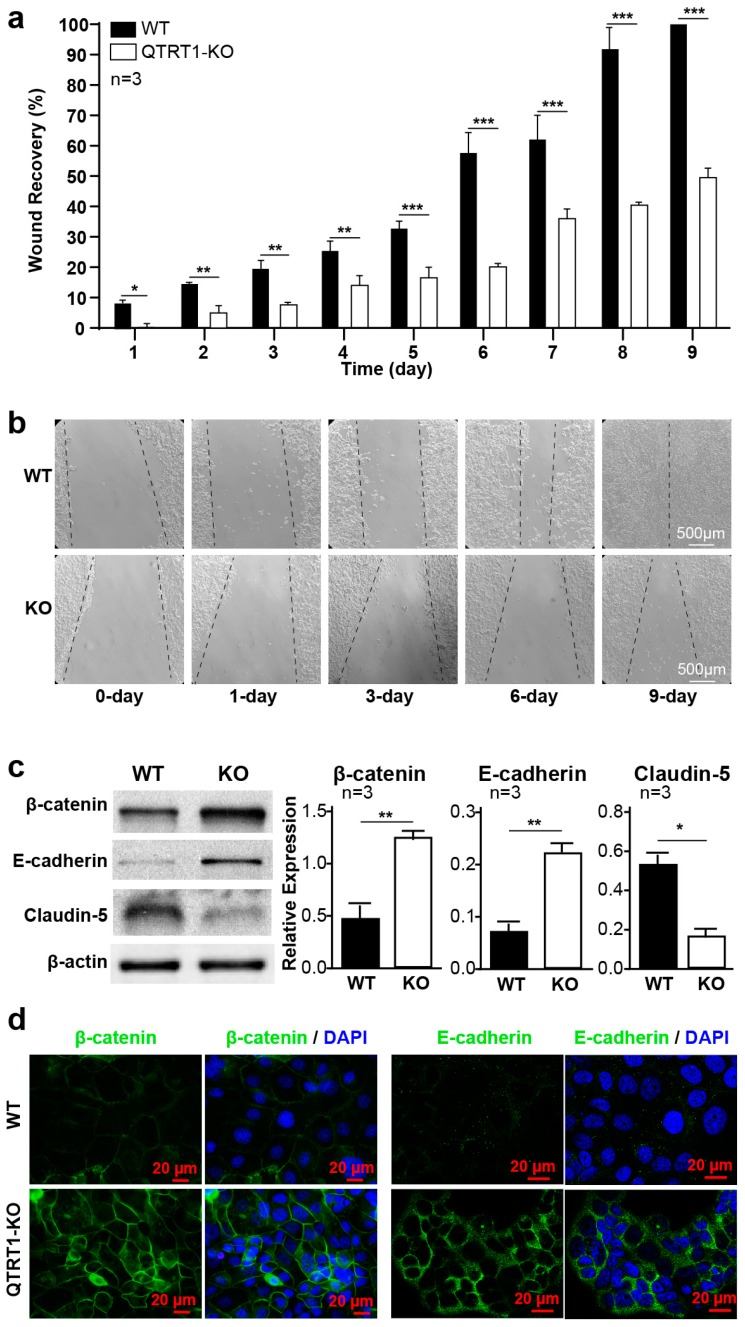
Knockout of QTRT1-suppressed cell migration and altered cell adhesion and tight junctions. (**a**) Wound healing analysis of wildtype and QTRT1-KO MCF7 cells was shown as the percentage of scratch closure at day timepoints. Mean ± SD, *n* = 3; * *p*-value < 0.05, ** *p*-value < 0.01, *** *p*-value < 0.001, two-tailed Welch’s *t*-test. (**b**) Representative wound healing images were shown. Scale bar is 500 µm. (**c**) Western analysis of tight junction protein E-cadherin, β-catenin, and caludin-5 was performed on QTRT1 knockout and WT MCF7 cells. Mean ± SD, *n* = 3; * *p*-value < 0.05, ** *p*-value < 0.01, two-tailed Welch’s *t*-test. (**d**) E-cadherin and β-catenin immunofluorescence staining showing the protein expression in QTRT1-KO MCF7 cells compared with WT cells. Proteins and DNA were stained with mouse monoclonal anti-β-catenin/E-cadherin and anti-mouse Alexa Fluor 488 antibody and DAPI, respectively. Scale bar is 20 µm.

The adherent cells have the ability to migrate and heal wounds, like the re-epithelialization of a skin scratch. To investigate the role of QTRT1 in the migration of breast cancer cells, we investigated the wound healing ability of these cells. We found that cells with suppressed QTRT1 expression healed the wound area significantly slower than that of the WT cells (Figure 2a,b). QTRT1 knockout in MCF7 cells significantly reduced cell migration at all the timepoints post wound healing (Figure 2a,b). Three-day post wounding the cells, the WT MCF7 cells healed around 20% of the wound area, whereas the QTRT1-KO cells healed less than 10% of the wound area (*p* < 0.05). After 9 days, WT cells healed 100% of the wound area compared with around 40% in QTRT1-KO MCF7 cells (*p* < 0.01) (Figure 2a,b).

The suppression of cell proliferation and migration of QTRT1 deletion were confirmed with the knockdown of QTRT1 in MDA-MB-231 breast cancer cells (Figure S2c–g).

### 2.3. Knockout of QTRT1 Altered Cell Adhesion and Tight Junctions

Cell adhesion plays an essential role in regulation of fundamental cellular process, such as cell proliferation and migration [11]. To elucidate the mechanism of how suppressed QTRT1 expression leads to altered proliferation and migration of breast cancer cells, we investigated the expression of markers of cell adhesion proteins, including β-catenin and E-cadherin. E-cadherin is also known as an anticancer protein. We found that knockout of QTRT1 significantly increased expression of E-cadherin and β-catenin and in the QTRT1-KO MCF7 cells, compared with the WT cells (Figure 2c).

Tight junction proteins are the key players in epithelial barrier function. Claudin-5 is an important tight junction protein, which is highly expressed in breast cancer patients with high-risk metastasis and reoccurrence [12]. We found that QTRT1 knockout in MCF7 cells significantly reduced the protein expression of claudin-5 (Figure 2c). The increased expression of membranous β-catenin and E-cadherin in QTRT1-KO MCF7 cells compared with wildtype (WT) cells was further confirmed using immunofluorescence staining (Figure 2d). After QTRT1-KO, the membrane β-catenin level was enhanced, suggesting the increased cell adhesion among cells. These data indicated the important role of QTRT1 in regulating cell junction pathways.

We analyzed the codon usage of the major cadherin and claudin transcript isoform. For claudin-5 (CLDN5), all Tyr (8 codons), His (3), Asn (4), and Asp (6) codons end with C in the third position. For E-cadherin (CDH1), the third codon position is generally C-preferred: Tyr (24 codons, 63% C), His (13, 77% C), Asn (43, 49% C), and Asp (63, 65% C). In comparison, C-ending codon fractions in the human genome are 56% (Tyr), 58% (His), 53% (Asn), and 54% (Asp). Q-modified tRNAs increase decoding of both U- and C-ending codons [13,14]. Therefore, the codon usage preference alone may not explain our observed effect on the expression of these two proteins.

Using the WT and QTRT1-KD MDA-MB-231 breast cancer cells, we showed the QTRT1-induced alterations of β-catenin and claudin-5 (Figure S2h).

### 2.4. Knockout of QTRT1 Decreased Tumor Growth and Altered Tight Junctions Regulators In Vivo

**Figure 3:**
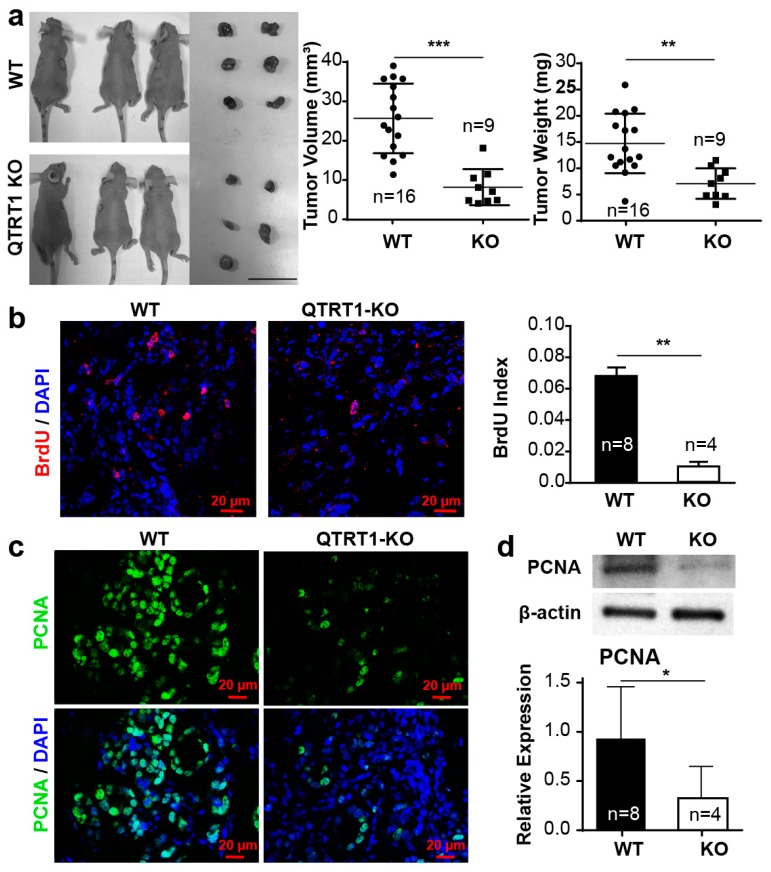

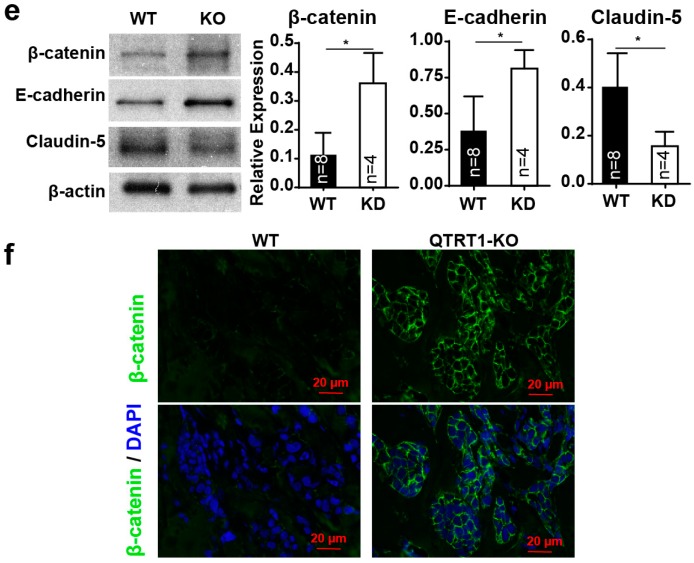
Knockout of QTRT1 suppressed tumor growth and altered tight junctions in tumors in vivo. (**a**) The WT and QTRT1-KO MCF7 cells were bilaterally injected into nude mice (*n* = 10 mice), and tumors were harvested 60 days post injection (WT: *n* = 16 tumors; KO: *n* = 9 tumors). The representative photograph of tumors in vivo and in vitro (left), tumor volume, and tumor weight were shown. The scale bar indicates 10 mm. Each circle represents an individual tumor. Mean ± SD, *n* = 16 or *n* = 9; ** *p*-value < 0.01, *** *p*-value < 0.001, two-tailed Welch’s *t*-test. (**b**) BrdU immunofluorescence staining and index analysis were performed on the tumors from nude mice injected with WT and QTRT1-KO MCF7 cells. BrdU was detected with sheep monoclonal anti-BrdU and anti-sheep Alexa Fluor 594 [20] antibody. The scale bar is 20 µm. Mean ± SD, *n* = 8 or *n* = 4; ** *p*-value < 0.01, two-tailed Welch’s *t*-test. (**c**) Immunocytochemical analysis of PCNA in tumors from nude mice. PCNA and DNA were stained with mouse monoclonal anti-PCNA and anti-mouse Alexa Fluor 488 antibody, and DAPI, respectively. The scale bar is 20 µm. (**d**) Western blot analysis of PCNA in tumors harvested from the mice injected with WT and QTRT1-KO MCF7 cells. The graph shows the band intensities of PCNA proteins. Mean ± SD *n* = 8 or *n* = 4; * *p*-value < 0.05, two-tailed Welch’s *t*-test. (**e**) Western blot analysis of tight junction regulators was performed on the tumors isolated from the nude mice model injected with WT and QTRT1-KO MCF7 breast cancer cells. The graphs show the relative expression of the target proteins detected by Western blot. Mean ± SD, *n* = 8 or *n* = 4; * *p*-value < 0.05, two-tailed Welch’s *t*-test. (**f**) Immunofluorescence staining of β-catenin was performed on tumors from both QTRT1-KO-injected mice and WT-injected mice. Proteins and DNA were stained with mouse monoclonal anti-β-catenin and anti-mouse Alexa Fluor 488 antibody and DAPI, respectively. Scale bar is 20 µm.

The changes of breast cancer cells in vitro have been replicated using direct orthotopic or heterotopic injection of cells into the mouse [15,16,17,18,19]. To investigate the impact of QTRT1 knockout on tumor outgrowth, the xenograft nude mouse model (*n* = 10 mice per group) was established by subcutaneous bilateral injection of QTRT1-KO or WT breast cancer cells. Individual mice may develop two, one, or zero tumors. We found that the total tumor number, tumor volume, and weight were significantly reduced in mice injected with QTRT1-KO MCF7 cells compared to the WT cells (*p* < 0.01) (Figure 3a). The BrdU index (number of cells stained with BrdU/number of total cells) was significantly lower in mice injected with QTRT1-KO cells than that of WT cells (*p* < 0.01) (Figure 3b), suggesting less cell proliferation in the QTRT1-KO cells in vivo. The expression of PCNA, another important proliferation regulator of cells, was also significantly decreased in tumors derived from QTRT1-KO MCF7 cells, compared with WT cells (Figure 3c,d).

As TJ proteins regulate several key signaling pathways in cancer process and development [21], we tested the markers of junction proteins in the tumors. Western blot analysis showed that the QTRT1-KO-induced tumors had significantly increased E-cadherin and β-catenin, but ablated expression levels of claudin-5 (Figure 3e). Immunofluorescence staining further showed increased β-catenin in tumors from QTRT1-KO injected mice, compared to that in WT MCF7 breast cancer cells (Figure 3f).

The suppression of tumor growth and alteration of TJ proteins in the tumors were also found in the QTRT1 knockdown MDA-MB-231 cells, using the xenograft nude mouse model (Figure S3). These data suggest that a knockdown of QTRT1 should be sufficient to induce all of these phenotypes.

### 2.5. Bacteria Were Detected in Tumors from the Nude Mice

**Figure 4:**
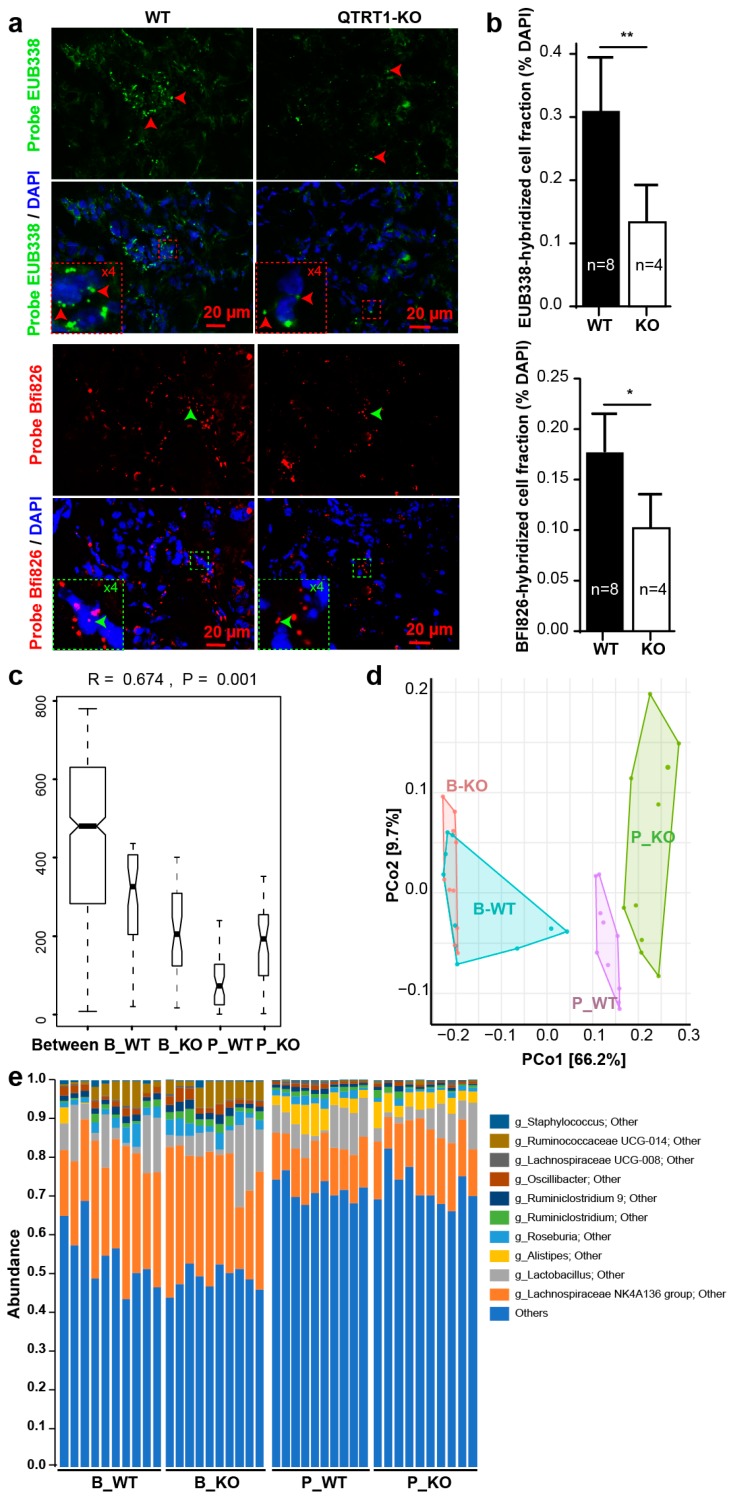
Altered microbiome in tumors and intestines of nude mouse model. (**a**) Fluorescence in situ hybridization [4] staining with DAPI, EUB338, and Bfi826 [20] of the tumors from the nude mice injected with wildtype and QTRT1-KO MCF7 breast cancer cells. Scale bar is 20 µm. (**b**) Relative bacteria staining was calculated as probe-hybridized cell / DAPI-stained cells. Mean ± SD *n* = 7 or *n* = 4; * *p*-value < 0.05, ** *p*-value < 0.01, two-tailed Welch’s *t*-test. (**c**) Plots of between and within means of Bray–Curtis dissimilarity. The analysis of similarity (ANOSIM) was performed on between (Between) and within groups of before QTRT1-KO cell injection (B_KO), before WT cell injection (B_WT), post QTRT1-KO cell injection (P_KO), and post WT cell injection (P_WT) based on the Bray–Curtis dissimilarity analysis. *n* = 10 per group. (**d**) The principal coordinates analysis (PCoA) plot of the mouse feces was produced to inspect the homogeneity of multivariate dispersions. The samples collected before (B_WT and B_KO) and post (P_WT and P_KO) cell injection were colored in the illustration. *n* = 10 per group. (**e**) Relative bacterial abundance in species level (g_ indicate genus level; the unidentified species were named with super level and other ) of feces collected from nude mice before (B_WT and B_KO) and post (P_WT and P_KO) injection of wildtype (WT) and QTRT1-KO MCF7 cells was shown with the top 30 species, and lower ones were grouped as “Others”. Species were colored using the key in the list on the right side. Each bar represents individual mice (*n* = 10 each group).

Two probes, EUB338 and Bfi826, were used for detecting total bacteria and *Butyrivibrio fibrisolvens*-related clones, which are in the gastrointestinal microbiome and ubiquitously present in the gastrointestinal tract of humans and animals, respectively. Fluorescence in situ hybridization with probes EUB338 and Bfi826 highlighted the bacteria replication in the tumors from the nude mice. The relative abundance of bacteria in tumor induced from WT cells was significantly higher than that of QTRT1-KO cells using both EUB338 and Bfi826 probes (*p* < 0.01 and *p* < 0.05, respectively) (Figure 4a,b). The bacteria’s existence in the tumors was confirmed with the tumor samples from the mice challenged with QTRT1-KD and WT MDA-MB-231 cells (Figure S4a).

### 2.6. Altered Gut Microbiome in the Nude Mice Injected with WT MCF7 Cells

We investigated whether gut bacteria have been changed in the nude mice injected with WT MCF7 cells or QTRT1-KO cells, using the data of 16S rRNA gene sequencing. We focused on hypothesis testing of two diversity measures: alpha diversities (Chao 1 and Shannon diversity) and beta diversity (Bray–Curtis dissimilarity). The analysis of the bacterial 16s rRNA gene sequencing showed that the intestinal microbiome community was different between the mice injected with WT and QTRT1-KO cells (Figure 4c–e; Figure S2).

Alpha diversity focuses the structure of a microbiome community to its richness (number of taxonomic groups), evenness (distribution of abundances of the groups), or both. The Chao 1 richness of bacteria at the species level was statistically significantly different between MCF7-WT injected mice and MCF-KO injected animals (*p* = 0.0112). Meanwhile, for the QTRT1-KO animals, the Chao 1 measure values of mice post-injection (mean ± SD: 203.8 ± 27.2; median: 209.8) was markedly increased, compared to pre-injection (mean ± SD: 162.1 ± 30.2; median: 151.3) with the false discovery rate (FDR) = 0.0106 (Benjamini–Hochberg method adjusted *p*-value). The similar increase in bacterial species was also found in QTRT1-WT mice between post-injection (mean ± SD: 181.1 ± 20.9; median: 179.7) and pre-injection (mean ± SD: 170.2 ± 28.1; median: 160.9) (FDR = 0.3153).

There were significant changes between MCF7-WT and MCF7-KO groups at the bacterial species level (*p* = 0.0002). The Shannon values of bacteria species was statistically decreased (FDR = 0.0028) post MCF7-KO cancer cell injection (mean ± SD: 2.1 ± 0.3; median: 2.1), compared to before injection (mean ± SD: 2.5 ± 0.1; median: 2.5). The same trend was found in the animals post-injection of MCF7-WT cells (mean ± SD: 2.1 ± 0.2; median: 2.1) and pre-injection (mean ± SD: 2.4 ± 0.2; median: 2.4) (FDR = 0.0099).

Beta diversity is to show differences or similarities among samples. We performed a permutational multivariate ANOVA (PERMANOVA) to detect the differences of Bray–Curtis dissimilarity among groups. The breast cancer cell injected groups had significantly different variations between WT MCF7 and QTRT1-KO groups (*p* = 0.0028) (Figure 4c). Our data showed that the groups accounted for 76% variations of the Bray–Curtis dissimilarities (*p* = 0.01) (Figure 4c). We then used a nonparametric procedure analysis of similarity (ANOSIM) based on a permutation test for analyzing among- and within-group similarity. ANOSIM further confirmed that the compositional dissimilarity of post-injection of WT MCF7 cells was significantly decreased compared with that before injection, but not of QTRT1-KO cells (*p* = 0.001). Principal coordinate analysis (PCoA) was used for the visualization of Bray–Curtis dissimilarity. In Figure 4d, PCoA showed that before injection, the ranges of the dissimilarities between WT and KO mice overlapped, whereas the ranges of bacterial dissimilarities increased in mice post injection.

Several core bacteria were markedly changed in mice post cell injection, such as *Lachnospiraceae*, *Lactobacillus,* and *Alistipes* (Figure 4e; Figures S5 and S6). The butyrate-producing genera *Lachnospiraceae* and *Ruminococcaceae*, where the genus could enhance producing butyric acid to protect the body from colon cancer [22] and triple-negative breast cancer (TNBC) [23], were found suppressed in the mouse model post breast cancer cell injection. The proportions of *Lactobacillus*, one of the common colonic microbiota, are frequently correlated with intestinal diseases like Crohn’s disease [24], obesity, type 2 diabetes, and cancer [25], and breast cancer tumor development [26] was found decreased as well. We also found a change in abundance of *Alistipes*, which may have disease-promoting activities with gut diseases and relatively high incidence in the nipple aspirate fluid from people with breast cancer [27,28,29].

The 16S rRNA gene sequencing was also performed using the fecal samples from mice injected with the MDA-MB-231 cells (Figure S4b–d). We observed altered microbiome in tumors and intestines of nude mice injected with MDA-MB-231 breast cancer cells. Microbial community was different between the mice injected with WT and QTRT1-deficient MDA-MB-231 cells. Our data demonstrated that the bacterial species level between WT and QTRT1-deficient groups had differences in alpha and beta diversities, which are potentially associated with breast cancer status and progression.

The microbial alteration was confirmed by real-time PCR with primers targeting 16S rRNA genes of *Lactobacillus*, *Lachnospiraceae,* and *Butyrivibrio fibrisolvens* [30,31,32] (Figure S6). In the colonic feces from the MCF7 injected mice, the relative abundance of *Lachnospiraceae* was significantly altered between the compared groups (*p* < 0.05) (Figure S6).

### 2.7. Inflammation Enhanced the Bacteria in Tumors of Nude Mice

**Figure 5:**
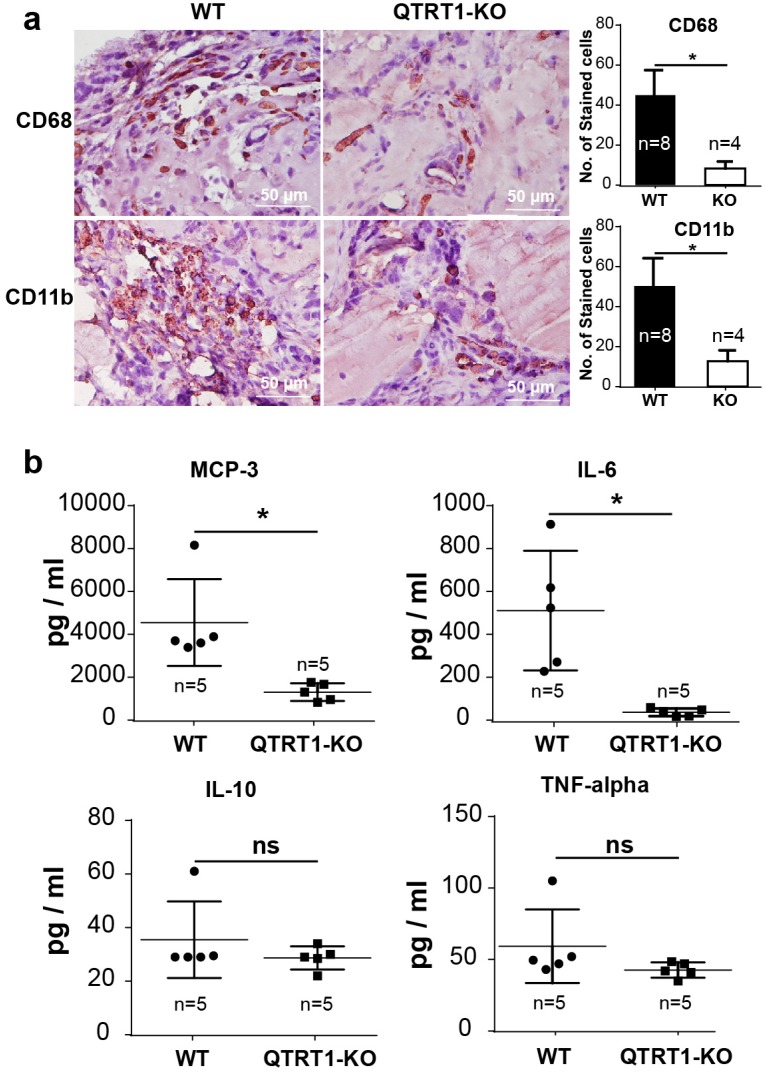
Inflammation regulators in tumors and plasma of nude mice. (**a**) Immunostaining of inflammation-related cells in tumors from nude mice with anti-CD68 and anti-CD11b. The number of stained cells were calculated in each visual filed. Scale bar is 50 µm. Mean ± SD, *n* = 8 or *n* = 4; * *p*-value < 0.05, two-tailed Welch’s *t*-test. (**b**) Mouse cytokine levels in the plasma of nude mice injected with WT and QTRT1-KO MCF7 breast cancer cells were determined by multiplex bead-based assays for 26 cytokines and chemokines. Mean ± SD, *n* = 5; * *p*-value < 0.05, ns means no significance.

Based on the results above, we further hypothesized that inflammation may lead to increased bacteria population in tumors. Cancer-related inflammation facilitates unlimited replicative potential, independence of growth factors, resistance to growth inhibition, escape of cell death, enhanced angiogenesis, and tumor extravasation [33,34]. Dysfunction of microbiome (dysbiosis) and bacterial metabolites could impact the tumor microenvironment and enhance chronic inflammation, thus affecting tumor growth and further stimulating bacterial multiplication [35]. The CD68-positive and CD-11b-positive inflammation-related cells were immunostained in the tumors from nude mice injected with WT or QTRT1-KO breast cancer cells, indicating an immune response inside the tumors (Figure 5a). The number of CD68-positive and CD11b-positive cells in the tumors from the mice injected with WT MCF7 cells was significantly higher than that of QTRT1-KO cells (*p* < 0.05) (Figure 5a). We further investigated the cytokines and chemokines levels in the plasma of the studied animals. MCP-3, the chemotactic gradients implicated in the process of inflammation, and interleukin 6 (IL-6), a pro-inflammatory cytokine, were found markedly increased in the mice injected with WT cells, compared with that of QTRT1-KO cells (Figure 5b). We did not find significant changes of IL-10 or TNF-α (Figure 5b).

### 2.8. Disrupted Tight Junctions in the Colon of Mice Injected with WT MCF7 Cells

**Figure 6:**
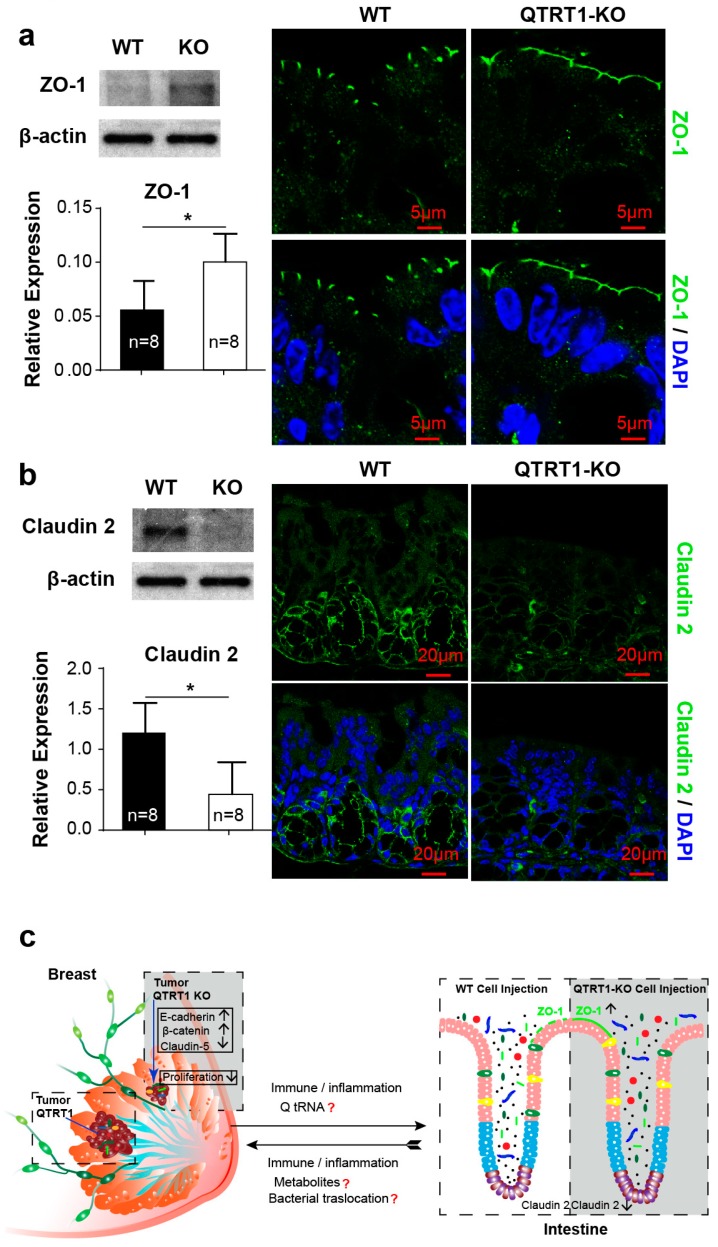
Altered tight junction proteins in the colon of a nude mouse model. Western blot analysis and immunofluorescence staining of ZO-1 (**a**) and claudin-2 (**b**) were performed on the colons of the nude mice injected with WT and QTRT1-KO MCF7 breast cancer cells. Proteins and DNA were stained with rabbit monoclonal anti-ZO-1/ mouse monoclonal anti-claudin-2 and anti-mouse Alexa Fluor 488 antibody, and DAPI, respectively. Scale bar is 5 µm in (**a**) and 20 µm in (**b**). The graphs show the relative expression of the target proteins detected by Western blot. Mean ± SD, *n* = 8; * *p*-value < 0.05, two-tailed Welch’s *t*-test. (**c**) The working model of QTRT1/Q-tRNA modification regulating gut-tumor-microbiome axis in breast cancer. Knockout of QTRT1 inhibited beast tumor proliferation and growth by altering the expressions of claudin-5, E-cadherin, and β-catenin in breast and changed the microenvironment of tumors. Meanwhile, the altered expression of intestinal junction proteins (claudin-2 and ZO-1) and altered bacterial community were found in the colon of mice with breast cancer cells. Microbiome-dependent tRNA Q-modifications regulate tumor growth and recruit microbiome to breast tumors. It is still unknown what the roles are of Q tRNA and metabolites in the process, and whether there is bacterial translocation from the intestine to the breast.

Intestinal tight junction [36] proteins, including claudins and zona occludens (e.g., ZO-1), have an important role in maintaining the integrity of the gut epithelium. Claudin-5 and claudin-2, both belonging to the claudin family, have totally different functions and tissue specificity in breasts and intestine. Claudin-5 is highly expressed in breast cancer patients with high-risk metastasis and reoccurrence. It was examined to verity the impact of QTRT1 knockout on the breast cell proliferation and migration (Figure 3). Claudin-2 is a leaky protein regulating intestinal permeability [37,38]. Dysfunction of intestinal TJs, including changes in protein distribution and expression level, could result in increased gut permeability and chronic inflammation [21]. Leaky gut is also known to further enhance dysbiosis and inflammation [37,39]. Thus, we investigated the intestinal TJs of the nude mice that were injected with WT or QTRT1-KO MCF7 cells. We found that the expression of the TJ protein ZO-1 was significantly reduced in the colon of nude mice injected with WT cells (Figure 6a). The structure disruption of ZO-1 was visualized in the colon of WT cell injected mice, whereas the ZO-1 distribution was intact in the colon of mice injected with QTRT1-KO cells (Figure 6a). Claudin-2 is considered a leaky protein that increases the intestinal permeability [37,38]. Interestingly, we found a significant increase of claudin-2 in the colon of WT cell injected mice, suggesting enhanced leaky gut (Figure 6b). In contrast, the colons of mice injected with QTRT1-KO cell showed significantly reduced claudin-2 (Figure 6b).

## 3. Discussion

In the current study, we demonstrated that QTRT1 plays a crucial role in breast cancer cell proliferation and growth both in vitro and in vivo. The proliferation and collective migration of QTRT1-KO MCF7 breast cancer cells were markedly suppressed, compared with its parent cells. Meanwhile, QTRT1 knockout leaded to changes in the expression of E-cadherin, β-catenin, and claudin-5, which are important cell junction proteins and facilitate cells adhesin formation. These differences were further confirmed in the xenograft nude mouse model in vivo. Furthermore, we found several core bacteria, such as *Lachnospiraceae*, *Lactobacillus,* and *Alistipes,* markedly altered in mice post injection with breast cancer cells. QTRT1 deficiency cells had reduced relative abundance of bacteria in tumors, compared to wildtype cell-induced tumors. All these changes in both cell culture and animals model levels were verified with another cell line of MDA-MB-231, which further strengthened our conclusions.

In our study, QTRT1 knockout or reduction could significantly increase the expression of β-catenin and change its location from inside the cells to the membrane, which participates in the formation, maintenance, and function of adherence junctions by linking cadherin to the actin cytoskeleton [40]. E-cadherin, one of the classical cadherins, which plays a crucial role in cell integrity and polarity, was also found upregulated in the QTRT1-KO cells. Thus, both the changes of E-cadherin and β-catenin have an impact on cell adhesion, combination, and polarity, and they further reduce the proliferation and migration of MCF7 cells after QTRT1 knockout. Claudins are major adhesion molecules in tight junctions and are strongly expressed in various cancers [41], and claudin-5 is highly expressed in breast cancer patients [15]. Interestingly, we found downregulation of claudin-5 in QTRT1-KO MCF7 cells. Suppressed cell proliferation in QTRT1 knockout contributed to marked reduction of the tumor growth, number, volume, and weight. The queuine-insertase complex, including QTRT1 (queuine tRNA-ribosyltransferase 1) and QTRTD1 (queuine-tRNA ribosyltransferase domain containing 1) subunits, has cytosolic proteins that could incorporate queuine base into tyrosyl, asparaginyl, aspartyl, and histidyl tRNAs. It is possible that modification of QTRT1-knockout on the Q-modified tRNAs could further impact cell gene transcription. As beta-catenin and tight junction proteins are important in cell gene transcription, it is reasonable that these proteins were changed after QTRT1 deficiency. However, more experiments should be performed to verify these possibilities. In the QTRT1-KO cells, we showed significantly reduced Q-modification levels (~25%). This incomplete elimination of Q-modification may be derived from either incomplete removal of potential QTRT1 splice isoforms in our cells, or the presence of an unknown gene or pseudogene that can also Q-modify tRNA. In any case, our KO cells did show a significant reduction of Q-levels, consistent with our experimental design. Q-modification most likely affects translation of a subset of genes. Since our primary goal was to investigate depletion of Q-modification impact on breast tumor growth, a detailed investigation on how translation of specific transcripts is affected will be a major goal in our follow-up study.

Interestingly, we found elevated levels of the microbiome in the tumors from WT-MCF7 cell injected mice. Increased inflammation and disrupted tight junctions in the colon of mice injected with WT MCF7 cells may contribute to accelerated breast cancer development (Figure 6c). As previously reported, the human and animal breasts are not sterile, rather containing a diverse and unique bacterial community. The breast microbiome is distinct from that of other body sites [42]. In vivo, we investigated the bacterial profile in the tumor in situ and in fecal samples. We detected the existence of bacteria in the tumors grown in the xenograft nude mice injected with QTRT1-KO and WT MCF7 cells. Meanwhile, we found that the enriched bacteria in tumors from WT MCF7 cells correlated with increased inflammation inside the tumors. Our data suggest bacterial replication in the tumor is associated with the changed microenvironment. Although it is still unclear whether the microbial differences are the consequences or causes of breast cancer or other breast diseases, the changes in the composition of breast microbiota could contribute to disease development and progression [42]. Similarly, our findings indicate that the QTRT1-associated altered microbiome and microenvironment may contribute to tumor growth.

The source of the bacteria in tumors may shed light on breast tumor development. *B. fibrisolvens* belongs to the genus *Butyrivibrio* and is part of the gastrointestinal microbiome of mammals and ubiquitously present in the gastrointestinal tract of many animals, including humans and mice [43]. These bacteria inside the tumors could be translocated from the intestine directly. Indeed, the intestinal lumen and outer mucus layer are the location of an overwhelming majority of gut bacteria, but it seems reasonable to envisage that a very small but significant minority of bacteria could occasionally breach the intestinal epithelium and quickly arrive at other organs or tissues, especially in the nude mice, which are immune deficient, used in our study. Recently, more and more data support the transfer of intestinal bacterial products and bacteria translocation directly or indirectly in some diseases, such as liver disease [44,45]. This hypothesis may be supported by the redistribution of ZO-1 and increased leaky protein claudin-2 in the colon of nude mice, which is related with intestinal permeability [46]. Tight junction proteins are key players in epithelial barrier function in inflammatory bowel disease [46,47,48,49,50]. Furthermore, the gut microbiome community of the mouse model markedly changed after breast cancer cell injection and tumor growth, compared with pre-injection, based on 16S ribosomal RNA gene amplification data. Also, taxonomic richness and diversity differences were also found between the mice injected with WT and KO cells. Intestinal dysbiosis, especially in the mice injected with WT cells, should further enhance dysfunctions in intestine. Therefore, the alteration of TJ proteins and microbiome components in the intestine should enhance bacterial translocation by regulating intestinal bacterial interactions with the mucosal surface, which may support bacterial translocation from the intestine to other locations or tumors. Increased cytokines and chemokines in the plasma of mice injected with WT MCF7 cells also support the idea that inflammation and leaky gut promote bacterial dysbiosis [51] and bacterial translocation.

Our data on the reduced butyrate-producing genera *Lachnospiraceae* and *Ruminococcaceae* in the mice with tumor growth support the role of intestinal dysbiosis in promoting breast cancer. Bacteria that enhance producing butyric acid are known to protect the body from colon cancer [22] and triple-negative breast cancer (TNBC) [23]. Intestinal *Lactobacillus* are frequently correlated with diseases, like Crohn’s disease [24], obesity, type 2 diabetes, cancer [25], and breast cancer tumor development [26]. We also found *Lactobacillus* decreased in mice with injection of breast cancer cells. Interestingly, we found a change in the abundance of *Alistipes*, which may promote digestive diseases, and relatively high incidence in the nipple aspirate fluid from people with breast cancer [27,28,29]. The potential origin of part of the breast tissue microbiome may be translocated from the gastrointestinal tract in addition to the skin via the nipple-areolar orifices [52]. Our data suggest the potential translocation of the gut microbiome to breast tumors.

The knockout of QTRT1 in MCF7 cells could impact the cell proliferation ability, which influences the microenvironment of the tumors and could feedback to affect the bacterial multiplication in the tumor. Moreover, the inflammation investigated in the tumors with related biomarkers (CD68 and CD11b) may be related with the bacterial growth in the tumors and microenvironment modification. More studies need to be done to investigate the relationship between the microbiome in tumors and tumor growth, and the source of these microorganisms. Another hypothesis was that the bacteria found in the tumors might be transferred from the skin, which touches the surrounding feces in the mouse cage. In the future, studies are also needed to understand the mechanisms of how QTRT1 interacts within TJ proteins to influence cell proliferation and tumor growth.

## 4. Methods

### 4.1. Regents and Cell Lines

The monoclonal antibody to human QTRT1 was purchased from Santa Cruz Biotechnology (Dallas, TX, USA). Monoclonal antibodies of β-catenin and E-cadherin from BD Transduction (San Jose, CA, USA) and β-actin were purchased from Sigma-Aldrich (St. Louis, MO, USA). Monoclonal antibodies of PCNA and CD11b (Integrin αM) were purchased from Santa Cruz Biotechnology (Dallas, TX, USA). Claudin-5 and claudin-2 mouse monoclonal antibodies and ZO-1 rabbit monoclonal antibody were purchased from Thermo Fisher Scientific (Rockford, IL, USA). CD68 rabbit polyclonal antibody was purchased from Abcam (Cambridge, MA, USA). All chemicals were purchased from Sigma-Aldrich unless otherwise stated. The breast cancer cell lines, including MCF7 and MDA-MB-231, were provided by Dr. Tao Pan’s lab at the University of Chicago.

### 4.2. Transfection and Selection

The highly aggressive breast cancer cell line human MCF7 cells were cultured in a 6-well tissue culture plate in antibiotic-free MCF7 cell growth medium (Eagle’s minimum essential medium with 10% FBS, 10 µg/mL bovine insulin, and 10 nM β-estradiol; Invitrogen, Carlsbad, CA, USA) to 70%–80% confluence. Cells were transfected with 2 µg of QTRT1 Double Nickase Plasmid (sc-413456-NIC), 10 µL LTX Lipofectamine, and 2.5 µL PLUS Reagent (Invitrogen) per well (manufacturer’s protocol). The plasmid encodes a D10A mutated Cas9 nuclease and a unique, target-specific 20-nt guide RNA (gRNA), which has greater specificity of gene knockout than the CRISPR/Cas9 KO plasmid counterpart. QTRT1 Double Nickase Plasmid-derived puromycin resistance gene was used for positive selection of transfected cells. Cells were selected with 1 µg/mL puromycin for 5 days when further cell death was not observed. After selection, cells were collected and serial diluted onto four 96-well plates. Single cells were expanded to obtain individual clones for further study. The culture of MDA-MB-231 cells was as described before [53], and transfection and selection were as described above.

### 4.3. Clone Validation

Individual clones after expansion to 6-well plate format were collected for clone validation. Cells were lysed in CelLytic™ M Lysis Reagent (Sigma-Aldrich, St. Louis, MO, USA) with a protease inhibitor cocktail (ThermoFisher, Waltham, MA, USA) overnight at 4 °C. The lysate was centrifuged at 16,000 g for 30 min, and 10 µg of supernatant was fractioned on 4–20% SDS PAGE gels, transferred to nitrocellulose, and screened by Western blot with QTRT1 antibody (Santa Cruz, CA, USA; sc-398918). This antibody (Santa Cruz, CA, USA; sc-398918) is specific for amino acids 111-136, exon 3, and a 5′ fragment of exon 4. QTRT1Genomic DNA was isolated from edited clones and nonedited MCF7 control cells with DNAzol (ThermoFisher, Waltham, MA, USA) and tested with PCR using QTRT1-specific primers in exon 1 (F1: 5′ end of the exon1: GGCGGGAGCAGCTACCCA) and intron downstream of exon 3 (Rev1_3: intron downstream of the exon 3: CCCGGCCTCAAGTGATCTTC). Clone validation of MD-MB-231 cells was performed as described above.

### 4.4. Cell Proliferation Assay

The WT and QTRT1 KO MCF7 cells were plated at a density of 1 × 10^4^ cells per well in triplicate. After two days of culture, proliferation was evaluated by the MTT Cell Proliferation Assay Kit (Thermo Fisher Scientific, Rockford, IL, USA) according to the product’s instructions [53]. Triplicate wells were counted for each time point, and the whole experiment was repeated three times. The evaluation of proliferation of WT MDA-MB-231 and QTRT1-KD (knockdown) MDA-MB-231 cells was performed as described above using the MTT Cell Proliferation Assay Kit.

### 4.5. Wound Healing Assay

Both wildtype (WT) and QTRT1-KO of MCF7 were plated on dishes (MatTek, Ashland, MA, USA) and cultured in humidified chambers with 5% CO_2_ at 37 °C. When cells were grown to confluency, the wounds were scratched and analyzed as described before [53]. Migration of cells into wounded areas was captured every day. The values were the means of three independent wound fields from three independent experiments (*n* = 3). As described above, the wound healing assay was also performed on the WT and QTRT1-KD MDA-MB-231 cells.

### 4.6. In Vivo Nude Mice Model

The 8-week old, specific-pathogen-free, female BALB/c Nude mice (*n* = 40) were purchased from Charles River Laboratories (Wilmington, MA). Animals were housed in the Biologic Resources Laboratory (BRL) at the University of Illinois at Chicago [54] and utilized in accordance with the UIC Animal Care Committee [54], the Office of Animal Care and Institutional Biosafety (OACIB) guidelines, and the animal protocol (number ACC 16–180). The same and consistent diet was provided to the animals throughout the experiment. The animals were separated as two groups including mice for MCF7-WT cell injection (*n* = 10) and mice for QTRT1-KO MCF7 cell injection (n = 10). The xenograft model was established by subcutaneous bilateral injection with 1.2 × 10^6^ cells in 200 µL of 50:50 Matrigel/PBS (phosphate-buffered saline) into the hind flank [55]. At 60 days post-tumor challenge, tumors and samples were harvested from the animals that were euthanized by IP injection of sodium pentobarbital (100 mg per kg body weight) followed by cervical dislocation. The weight of the tumors was scaled, and the tumor volume (V) was calculated with caliper measurements using formulas V = (W^2^ × L)/2 [56]. BrdU staining was performed as previously described [57]. Protein expression in tumor tissues was detected by immunofluorescence staining and Western blot as described below. Wildtype and QTRT1-KD MDA-MB-231 cells (7.5 × 10^6^) were also analyzed using the BALB/c Nude mice (*n* = 40), as described above, except that the tumors and samples were harvested at 30 days post cell injection.

### 4.7. Western Blot Analysis

Breast tissue was lysed in stocked lysis buffer [53]. Cultured cells were rinsed twice in ice-cold Hanks’ balanced salt solution (Sigma-Aldrich, Saint Louis, MO, USA) and lysed in protein loading buffer then followed by sonication (Branson Sonifier, Danbury, CT, USA) and centrifugation [53]. The target proteins were detected by special primary antibody (1:1000) followed by secondary antibody conjugated to horseradish peroxidase at 1:5000 dilution. The blots were visualized by ECL chemiluminescence (Thermo Scientific, Rockford, IL, USA). All experiments were performed 3–5 times. Western blot bands were quantified using image analyzer (ImageJ, NIH, Bethesda, MD, USA).

### 4.8. The Northern Blot Analysis

The Northern blot method using 3-acrylamidophenylboronic acid (APB) gels to measure tRNA Q-modification levels was described in detail previously [8]. Oligonucleotide probe sequences were as follows:

tRNA^His^:5′-TGCCGTGACTCGGATTCGAACCGAGGTTGCTGCGGCCACAACGCAGAGTACTAA CCACTATACGATCACGGC;
tRNA^Asn^:5′-CGTCCCTGGGTGGGCTCGAACCACCAACCTTTCGGTTAACAGCCGAACGCGCTA ACCGATTGCGCCACAGAGAC.

### 4.9. Immunofluorescence and Confocal Imaging

Wildtype and knockout/knockdown cells were plated on fibronectin-coated glass coverslips and cultured with medium described above to monolayer in humidified chambers with 5% CO_2_ at 37 °C. The cells were immunostained and imaged as described before [53]. All experiments were performed multiple times using independent biological replicates.

Fresh tumors were fixed in 10% neutral buffered formalin followed by paraffin embedding. For immunofluorescence staining [58], slides were incubated in 5% bovine serum albumin (BSA) with 0.1% goat serum in PBS for 1 h at room temperature to reduce nonspecific background. The samples were incubated overnight at 4 °C with primary antibody at 1:100 dilution. The sections were then incubated with secondary antibodies and DAPI for 1 h at room temperature, and they examined with confocal microscope as described before [59].

For fluorescence in situ hybridization [4] staining, fresh tumor tissue was fixed with 10% neutral-buffered formalin and embedded in paraffin. The sections were treated successively with 0.2 M HCl, 1 M NaSCN, and 4% pepsin, then hybridized with the EUB338 probe (5′-GCTGCCTCCCGTAGGAGT-3′) for all bacteria and Bfi826 (5’-ATGGCACCCAACACCTAG-3’) for *Butyrivibrio fibrisolvens*-related clones [60] in hybridization buffer (0.9 M NaCl, 0.02 M Tris-HCl, pH 7.6, 0.01% sodium dodecyl sulfate) at 37 °C overnight. Slides were then incubated for 10 min with FISH (fluorescence in situ hybridization) washing buffer (0.3M NaCl, 0.03 M Trisodium citrate) preheated to 45 °C. Slides were imaged using a confocal microscope as described above. All experiments were performed 3–5 times.

### 4.10. Immunohistochemistry

Tissues were fixed in 10% neutral-buffered formaldehyde for 2 h, transferred into 70% ethanol, and processed the next day by standard techniques. Immunohistochemistry for inflammation cells was performed on paraffin-embedded sections (4 µm) of tumors. Briefly, the paraffin sections were baked in an oven at 56 °C for 30 min. The sides were deparaffinized and rehydrated in xylene, followed by graded ethanol washes at room temperature. Antigen retrieval was achieved by boiling the slides in a microwave oven with 0.01 M, pH 6.0 sodium citrate buffer. Sides were then incubated in hydrogen peroxide (3% H_2_O_2_ in PBS) for 10 min at room temperature, followed by incubation in 5% fetal bovine serum/PBS for 1 h. The inflammation cells were stained with polyclonal CD 68 antibody (Abcam, Cambridge, MA, USA) and monoclonal CD 11b antibody (Santa Cruz, Dallas, TX, USA).

### 4.11. Luminex Immunoassays

The 26 cytokines and chemokines of plasma samples were analyzed using the mouse cytokine and chemokine magnetic 26-ple ProcartalPlex Panel 1 (Thermo Fisher Scientific, Waltham, MA, USA) with 25 µL of samples according to the manufacturer’s instructions using a 2 h incubation at room temperature. Samples were read on a MAGPIX^TM^ system platform (Millipore Sigma, Burlington, MA, USA).

### 4.12. Microbial Sampling and Sequencing

The day before cell injection, fresh feces were collected directly from the mouse into the sterile tubes. At the end of the experiment, fresh fecal samples were isolated from the colon and placed into the specially prepared sterile tubes. The samples were kept at low temperature with dry ice and were sent to the UIC RRC (University of Illinois at Chicago Research Resources Center, Chicago, IL, USA) for genomic sequencing. The DNA of samples was extracted using the DNeasy Power Fecal Kit (Qiagen, Hilden, Germany) based on the manufacturer’s instructions with a slight modification. The samples were heated at 65 °C for 10 min before homogenizing with FastPre-24 5G bead-beating device (MP Biomedicals, Solon, OH, USA) at 6 m/s for 40 s. The workflow for preparing samples for next-generation amplicon sequencing contains two independent PCR steps. The first stage PCR amplification was performed using primers containing CS1 and CS2 linkers (CS1_341F: 5′-ACACTGACGACATGGTTCTACAGTGCCAGCMGCCGCGGTAA-3′; CS2_806R: 5′-TACGGTAGCAGAGACTTGGTCTGGACTACHVGGGTWTCTAAT-3′) to the V3–V4 variable region of the 16S rRNA gene, while the second stage of PCR amplification was performed on the first stage of PCR products using the Fluidigm Access Array barcoded primers. The 16S rRNA gene metagenomic sequencing was performed using MiSeq according to the Illumina protocol.

### 4.13. Sequencing Bioinformatics

After DNA sequencing, all possible raw paired-end reads were evaluated and merged using the PEAR (Paired-End reAd mergeR) software (http://www.exelixis-lab.org/web/software/pear.) [61]. To filter out low-quality reads, quality trimming was processed based on quality threshold (*p* = 0.01) and length parameters (minimum length = 225). The adapter/primer sequences and ambiguous nucleotides were trimmed from reads. Sequencing chimera filtering was carried out using bot reference and algorithm methods, with sequences that passed both these being retained and operational taxonomic units (OTUs) selected based on a 97% similarity representing each OTU, and both methods utilized the USEARCH (http://www.drive5.com/usearch) algorithm and the reference database silva_132_16S.97 [62]. Then, OTUs were annotated using the USEARCH (http://www.drive5.com/usearch) algorithm as compared with a reference database (silva_132_16S.97) [63]. An OTU table was generated from these results, and alpha and beta diversity metrics were generated, calculated, and visualized as described below.

Initially, 1,314,538 reads were assembled from the source sequencing data. After trimming, the number of reads diminished to 1,290,241, and then after chimera checking it reduced to 1,211,340, which was used as the operational OTUs in the alignment. The OTUs ranged from 54,043 to 65,772 for the individual sample, with a mean of 60,567. Taxonomic assignments of the microbiomes of the studied samples were obtained with the OTU data. On the phylum level, Bacteroidetes and Firmicutes accounted for the major part of the microbial population in all samples (96.4%–99.4%).

### 4.14. Statistical Analysis

Data shown in the bar figures were the average values from at least three independent experiments with the mean ± SD. All statistical tests were two-sided. It was considered statistically significant with *p*-value < 0.05. One, two, and three asterisks on the bars in the figures represent *p*-values < 0.05, < 0.01, and < 0.001, respectively. To investigate the dynamic effects of QTRT1-KO/KD on cell migration in breast cancer cells, the group effects from 1 to 9 days (Figure 2a, Figure S2f) were tested using generalized linear mixed models (GLMMs). The GLMMs were performed using SAS version 9.4 (SAS Institute, Inc., Cary, NC, USA); other statistical analyses were conducted by GraphPad Prism 5 (GraphPad Software, Inc., La Jolla, CA, USA).

The alpha and beta diversity indices including Shannon diversity [64,65], Chao1 richness [66], and Bray–Curtis dissimilarity [67] were calculated and visualized to show the microbial abundance and diversity. Bar plot and heatmap were used to visualize the identified “core” bacteria, which were defined as the set of species shared by (almost) all individuals with 0.1% relative abundance in more than 50% of the samples. Principal coordinate analysis (PCoA) plots were used for the visualization of Bray–Curtis dissimilarity [68]. To detect the differences of Bray–Curtis dissimilarity among groups, PERMANOVA was performed, followed by a variance homogeneity assumption testing to ensure the reliability of the PERMANOVA results. Then, a nonparametric procedure analysis of similarity (ANOSIM) based on a permutation test was used for analyzing among- and within-group similarity. The latest version of R and R packages of ampvis2, microbiome, phyloseq, and vegan were used for microbiome data analyses as we did in our book using R software [69].

### 4.15. Availability of Data and Material 

The raw sequence data from 16S rRNA gene amplicon sequencing were submitted to NCBI SRA under BioProject accession number PRJNA599383 (https://www.ncbi.nlm.nih.gov/sra/PRJNA599383).

## 5. Conclusions

Conclusively, we have demonstrated the important functions of QTRT1/Q-tRNA modification in regulating development of breast cancer.. Knockout or reduction of QTRT1 could significantly suppress the proliferation and migration of breast cancer cells. The knockout of QTRT1 resulted in the loss of aggressiveness of cancer cells both in vitro and in vivo. Microbiome-dependent tRNA Q-modifications regulate breast tumor growth and recruit microbiome to breast tumors. We have identified the close relation between the microbiome and microenvironment in tumors and the intestine. Our findings provide new insights into manipulating QTRT1 as a potential gene in controlling tumor growth and development through the gut–microbiome–tumor axis.

## Supplementary Materials

The following are available online at https://www.mdpi.com/2072-6694/12/3/628/s1, Figure S1. Verification of QTRT1 knockout in MCF7 cells by sequencing and Northern blot. Figure S2. Knockdown of QTRT1 suppressed MDA-MB-231 breast cancer cell proliferation, migration, and altered cell adhesion and tight junctions. Figure S3. Knockdown of QTRT1 suppressed tumor growth and altered tight junctions in tumors in vivo. Figure S4. Altered microbiome in tumors and intestines of nude mouse model. Figure S5. Heatmap of the bacterial percentage abundance in the intestine of nude mice. Figure S6. Relative abundance of intestinal bacteria detected with real-time PCR.

## References

[1] Honda S., Loher P., Shigematsu M., Palazzo J.P., Suzuki R., Imoto I., Rigoutsos I., Kirino Y. (2015). Sex hormone-dependent tRNA halves enhance cell proliferation in breast and prostate cancers. Proc. Natl. Acad. Sci. USA.

[2] Guzman N., Agarwal K., Asthagiri D., Yu L., Saji M., Ringel M.D., Paulaitis M.E. (2015). Breast Cancer-Specific miR Signature Unique to Extracellular Vesicles Includes “microRNA-like” tRNA Fragments. Mol. Cancer Res..

[3] Dhahbi J.M., Spindler S.R., Atamna H., Boffelli D., Martin D.I. (2014). Deep Sequencing of Serum Small RNAs Identifies Patterns of 5’ tRNA Half and YRNA Fragment Expression Associated with Breast Cancer. Biomark. Cancer.

[4] Goodarzi H., Liu X., Nguyen H.C., Zhang S., Fish L., Tavazoie S.F. (2015). Endogenous tRNA-Derived Fragments Suppress Breast Cancer Progression via YBX1 Displacement. Cell.

[5] Fergus C., Barnes D., Alqasem M.A., Kelly V.P. (2015). The queuine micronutrient: Charting a course from microbe to man. Nutrients.

[6] Morris R.C., Brown K.G., Elliott M.S. (1999). The effect of queuosine on tRNA structure and function. J. Biomol. Struct. Dyn..

[7] Ames B.N. (2018). Prolonging healthy aging: Longevity vitamins and proteins. Proc. Natl. Acad. Sci. USA.

[8] Wang X., Matuszek Z., Huang Y., Parisien M., Dai Q., Clark W., Schwartz M.H., Pan T. (2018). Queuosine modification protects cognate tRNAs against ribonuclease cleavage. Rna.

[9] Muller M., Legrand C., Tuorto F., Kelly V.P., Atlasi Y., Lyko F., Ehrenhofer-Murray A.E. (2019). Queuine links translational control in eukaryotes to a micronutrient from bacteria. Nucleic Acids Res..

[10] Tuorto F., Legrand C., Cirzi C., Federico G., Liebers R., Muller M., Ehrenhofer-Murray A.E., Dittmar G., Grone H.J., Lyko F. (2018). Queuosine-modified tRNAs confer nutritional control of protein translation. EMBO J..

[11] Matsunaga T., Iyoda T., Fukai F. (2014). Adhesion-dependent cell Regulation via Adhesion molecule, integrin: Therapeutic application of integrin activation-modulating factors. Colloid and Interface Science in Pharmaceutical Research and Development.

[12] Sugimoto H., Nagahara M., Bae Y., Nakagawa T., Ishikawa T., Sato T., Uetake H., Eishi Y., Sugihara K. (2015). Clinicopathologic relevance of claudin 5 expression in breast cancer. Am. J. Clin. Pathol..

[13] Meier F., Suter B., Grosjean H., Keith G., Kubli E. (1985). Queuosine modification of the wobble base in tRNAHis influences ‘in vivo’ decoding properties. EMBO J..

[14] Zaborske J.M., DuMont V.L., Wallace E.W., Pan T., Aquadro C.F., Drummond D.A. (2014). A nutrient-driven tRNA modification alters translational fidelity and genome-wide protein coding across an animal genus. PLoS Biol..

[15] Di Cello F., Shin J., Harbom K., Brayton C. (2013). Knockdown of HMGA1 inhibits human breast cancer cell growth and metastasis in immunodeficient mice. Biochem. Biophys. Res. Commun..

[16] Ooms L.M., Binge L.C., Davies E.M., Rahman P., Conway J.R., Gurung R., Ferguson D.T., Papa A., Fedele C.G., Vieusseux J.L. (2015). The Inositol Polyphosphate 5-Phosphatase PIPP Regulates AKT1-Dependent Breast Cancer Growth and Metastasis. Cancer Cell.

[17] Stengel C., Newman S.P., Leese M.P., Thomas M.P., Potter B.V., Reed M.J., Purohit A., Foster P.A. (2015). The In Vitro and In Vivo Activity of the Microtubule Disruptor STX140 Is Mediated by Hif-1 Alpha and CAIX Expression. Anticancer Res..

[18] Yano S., Takehara K., Miwa S., Kishimoto H., Tazawa H., Urata Y., Kagawa S., Bouvet M., Fujiwara T., Hoffman R.M. (2016). In Vivo Isolation of a Highly-aggressive Variant of Triple-negative Human Breast Cancer MDA-MB-231 Using Serial Orthotopic Transplantation. Anticancer Res..

[19] Khaled M., Belaaloui G., Jiang Z.Z., Zhu X., Zhang L.Y. (2016). Antitumor effect of Deoxypodophyllotoxin on human breast cancer xenograft transplanted in BALB/c nude mice model. J. Infect. Chemother..

[20] Nicolas A., Kenna K.P., Renton A.E., Ticozzi N., Faghri F., Chia R., Dominov J.A., Kenna B.J., Nalls M.A., Keagle P. (2018). Genome-wide Analyses Identify KIF5A as a Novel ALS Gene. Neuron.

[21] Bhat A.A., Uppada S., Achkar I.W., Hashem S., Yadav S.K., Shanmugakonar M., Al-Naemi H.A., Haris M., Uddin S. (2018). Tight Junction Proteins and Signaling Pathways in Cancer and Inflammation: A Functional Crosstalk. Front. Physiol..

[22] Meehan C.J., Beiko R.G. (2014). A phylogenomic view of ecological specialization in the Lachnospiraceae, a family of digestive tract-associated bacteria. Genome Biol. Evol..

[23] Collard M., Austin N., Tallant A., Gallagher P. (2019). Muscadine Grape Extract Reduces Lung and Liver Metastasis in Mice with Triple Negative Breast Cancer in Association with Changes in the Gut Microbiome (P05-017-19). Curr. Dev. Nutr..

[24] Langille M.G., Zaneveld J., Caporaso J.G., McDonald D., Knights D., Reyes J.A., Clemente J.C., Burkepile D.E., Vega Thurber R.L., Knight R. (2013). Predictive functional profiling of microbial communities using 16S rRNA marker gene sequences. Nat. Biotechnol..

[25] Heeney D.D., Gareau M.G., Marco M.L. (2018). Intestinal Lactobacillus in health and disease, a driver or just along for the ride?. Curr. Opin. Biotechnol..

[26] Jafari S., Froushani S.M.A., Tokmachi A. (2017). Combined Extract of Heated 4T1 and a Heat-Killed Preparation of Lactobacillus Casei in a Mouse Model of Breast Cancer. Iran. J. Med. Sci..

[27] Mancabelli L., Milani C., Lugli G.A., Turroni F., Cocconi D., van Sinderen D., Ventura M. (2017). Identification of universal gut microbial biomarkers of common human intestinal diseases by meta-analysis. FEMS Microbiol. Ecol..

[28] Yang Y., Jobin C. (2017). Novel insights into microbiome in colitis and colorectal cancer. Curr. Opin. Gastroenterol..

[29] Chan A.A., Bashir M., Rivas M.N., Duvall K., Sieling P.A., Pieber T.R., Vaishampayan P.A., Love S.M., Lee D.J. (2016). Characterization of the microbiome of nipple aspirate fluid of breast cancer survivors. Sci. Rep..

[30] Frank J.A., Reich C.I., Sharma S., Weisbaum J.S., Wilson B.A., Olsen G.J. (2008). Critical evaluation of two primers commonly used for amplification of bacterial 16S rRNA genes. Appl. Environ. Microbiol..

[31] Kennedy N.A., Walker A.W., Berry S.H., Duncan S.H., Farquarson F.M., Louis P., Thomson J.M. (2014). The impact of different DNA extraction kits and laboratories upon the assessment of human gut microbiota composition by 16S rRNA gene sequencing. PLoS ONE.

[32] Zhang Y.-g., Wu S., Yi J., Xia Y., Jin D., Zhou J., Sun J. (2017). Target intestinal microbiota to alleviate disease progression in amyotrophic lateral sclerosis. Clin. Ther..

[33] Hanahan D., Weinberg R.A. (2000). The hallmarks of cancer. Cell.

[34] Hanahan D., Weinberg R.A. (2011). Hallmarks of cancer: The next generation. Cell.

[35] Song M., Chan A.T., Sun J. (2020). Influence of the Gut Microbiome, Diet, and Environment on Risk of Colorectal Cancer. Gastroenterology.

[36] Gallo V., Bueno-De-Mesquita H.B., Vermeulen R., Andersen P.M., Kyrozis A., Linseisen J., Kaaks R., Allen N.E., Roddam A.W., Boshuizen H.C. (2009). Smoking and Risk for Amyotrophic Lateral Sclerosis: Analysis of the EPIC Cohort. Ann. Neurol..

[37] Zhang Y.G., Wu S., Lu R., Zhou D., Zhou J., Carmeliet G., Petrof E., Claud E.C., Sun J. (2015). Tight junction CLDN2 gene is a direct target of the vitamin D receptor. Sci. Rep..

[38] Zhang Y.G., Wu S., Xia Y., Sun J. (2013). Salmonella infection upregulates the leaky protein claudin-2 in intestinal epithelial cells. PLoS ONE.

[39] Clapp M., Aurora N., Herrera L., Bhatia M., Wilen E., Wakefield S. (2017). Gut microbiota’s effect on mental health: The gut-brain axis. Clin. Pract..

[40] Terada N., Karim M.R., Izawa T., Kuwamura M., Yamate J. (2017). Immunolocalization of beta-catenin, E-cadherin and N-cadherin in neonate and adult rat kidney. J. Vet. Med. Sci..

[41] Jia W., Lu R., Martin T.A., Jiang W.G. (2014). The role of claudin-5 in blood-brain barrier (BBB) and brain metastases (review). Mol. Med. Rep..

[42] Fernandez M.F., Reina-Perez I., Astorga J.M., Rodriguez-Carrillo A., Plaza-Diaz J., Fontana L. (2018). Breast Cancer and Its Relationship with the Microbiota. Int. J. Environ. Res. Public Health.

[43] Rodriguez Hernaez J., Ceron Cucchi M.E., Cravero S., Martinez M.C., Gonzalez S., Puebla A., Dopazo J., Farber M., Paniego N., Rivarola M. (2018). The first complete genomic structure of Butyrivibrio fibrisolvens and its chromid. Microb. Genom..

[44] Chassaing B., Etienne-Mesmin L., Gewirtz A.T. (2014). Microbiota-liver axis in hepatic disease. Hepatology.

[45] Ma H.D., Wang Y.H., Chang C., Gershwin M.E., Lian Z.X. (2015). The intestinal microbiota and microenvironment in liver. Autoimmun. Rev..

[46] Lee S.H. (2015). Intestinal permeability regulation by tight junction: Implication on inflammatory bowel diseases. Intest. Res..

[47] Shang M., Sun J. (2017). Vitamin D/VDR, Probiotics, and Gastrointestinal Diseases. Curr. Med. Chem..

[48] Landy J., Ronde E., English N., Clark S.K., Hart A.L., Knight S.C., Ciclitira P.J., Al-Hassi H.O. (2016). Tight junctions in inflammatory bowel diseases and inflammatory bowel disease associated colorectal cancer. World J. Gastroenterol..

[49] Sun J., Kato I. (2016). Gut microbiota, inflammation and colorectal cancer. Genes Dis..

[50] Lechuga S., Ivanov A.I. (2017). Disruption of the epithelial barrier during intestinal inflammation: Quest for new molecules and mechanisms. Biochim. Et Biophys. Acta. Mol. Cell Res..

[51] Rosean C.B., Bostic R.R., Ferey J.C., Feng T.-Y., Azar F.N., Tung K.S., Dozmorov M.G., Smirnova E., Bos P.D., Rutkowski M.R. (2019). Pre-existing commensal dysbiosis is a host-intrinsic regulator of tissue inflammation and tumor cell dissemination in hormone receptor-positive breast cancer. Cancer Res..

[52] Hieken T.J., Chen J., Hoskin T.L., Walther-Antonio M., Johnson S., Ramaker S., Xiao J., Radisky D.C., Knutson K.L., Kalari K.R. (2016). The Microbiome of Aseptically Collected Human Breast Tissue in Benign and Malignant Disease. Sci. Rep..

[53] Zhang J., Li K., Zhang Y., Lu R., Wu S., Tang J., Xia Y., Sun J. (2019). Deletion of sorting nexin 27 suppresses proliferation in highly aggressive breast cancer MDA-MB-231 cells in vitro and in vivo. BMC Cancer.

[54] Aad G., Abbott B., Abbott D.C., Abdinov O., Abed Abud A., Abeling K., Abhayasinghe D.K., Abidi S.H., AbouZeid O.S., Abraham N.L. (2020). Search for Magnetic Monopoles and Stable High-Electric-Charge Objects in 13 Tev Proton-Proton Collisions with the ATLAS Detector. Phys. Rev. Lett..

[55] Kocaturk B., Versteeg H.H. (2015). Orthotopic injection of breast cancer cells into the mammary fat pad of mice to study tumor growth. J. Vis. Exp. Jove.

[56] Faustino-Rocha A., Oliveira P.A., Pinho-Oliveira J., Teixeira-Guedes C., Soares-Maia R., da Costa R.G., Colaco B., Pires M.J., Colaco J., Ferreira R. (2013). Estimation of rat mammary tumor volume using caliper and ultrasonography measurements. Lab Anim..

[57] Lu R., Wu S., Liu X., Xia Y., Zhang Y.G., Sun J. (2010). Chronic effects of a Salmonella type III secretion effector protein AvrA in vivo. PLoS ONE.

[58] Tack D.M., Marder E.P., Griffin P.M., Cieslak P.R., Dunn J., Hurd S., Scallan E., Lathrop S., Muse A., Ryan P. (2019). Preliminary incidence and trends of infections with pathogens transmitted commonly through food—Foodborne Diseases Active Surveillance Network, 10 U.S. sites, 2015–2018. Am. J. Transplant..

[59] Lu R., Wu S., Zhang Y.G., Xia Y., Zhou Z., Kato I., Dong H., Bissonnette M., Sun J. (2016). Salmonella Protein AvrA Activates the STAT3 Signaling Pathway in Colon Cancer. Neoplasia.

[60] Kong Y., He M., McAlister T., Seviour R., Forster R. (2010). Quantitative fluorescence in situ hybridization of microbial communities in the rumens of cattle fed different diets. Appl. Environ. Microbiol..

[61] Zhang J., Kobert K., Flouri T., Stamatakis A. (2014). PEAR: A fast and accurate Illumina Paired-End reAd mergeR. Bioinformatics.

[62] Glockner F.O., Yilmaz P., Quast C., Gerken J., Beccati A., Ciuprina A., Bruns G., Yarza P., Peplies J., Westram R. (2017). 25 years of serving the community with ribosomal RNA gene reference databases and tools. J. Biotechnol..

[63] Edgar R.C. (2010). Search and clustering orders of magnitude faster than BLAST. Bioinformatics.

[64] Shannon C.E. (1948). A mathematical theory of communication. Bell Syst. Tech. J..

[65] Shannon C.E., Weaver W. (1949). The Mathematical Theory of Communication.

[66] Chao A. (1984). Nonparametric estimation of the number of classes in a population. Scand. J. Stat..

[67] Bray J.R., Curtis J.T. (1957). An ordination of upland forest communities of southern Wisconsin. Ecol. Monogr..

[68] Xia Y., Sun J., Chen D.-G. (2018). Statistical Analysis of Microbiome Data with R.

[69] R Core Team (2019). R: A Language and Environment for Statistical Computing.

