# Supplementary material for: tRNA Queuosine Modification Enzyme Modulates the Growth and Microbiome Recruitment to Breast Tumors
Source: Cancers (Basel). 2020 Mar 9;12(3):628. doi: 10.3390/cancers12030628 (PMC7139606; doi:10.3390/cancers12030628)
Supplement: Supplementary file 1 [file cancers-12-00628-s001.zip › Supplementary File.pdf]

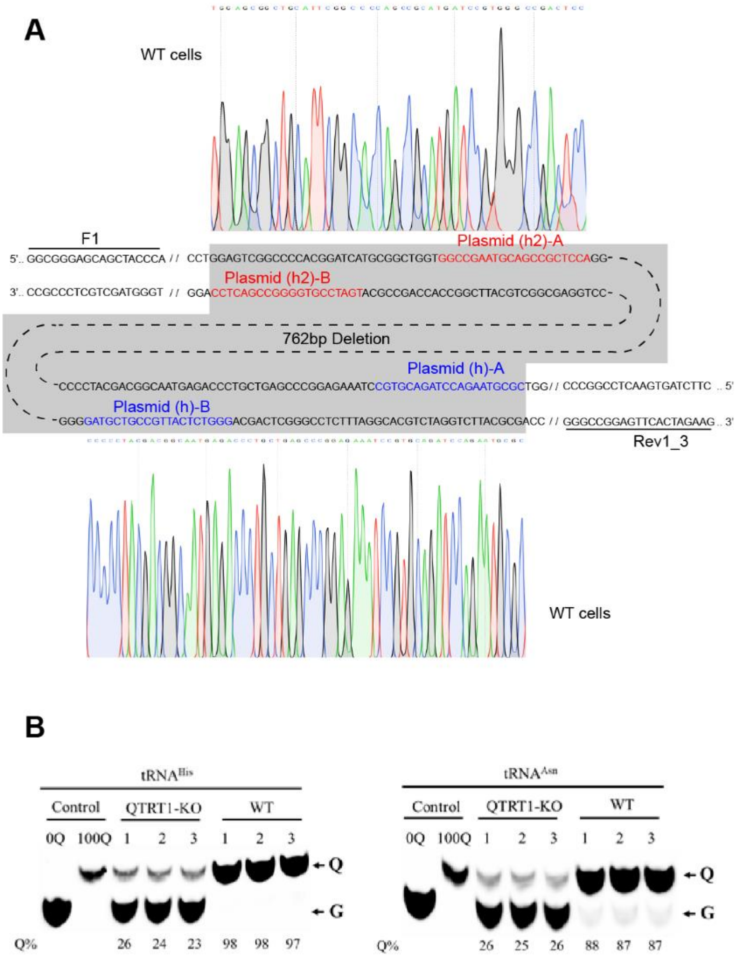

Figure S1. Verification of QTRT1 knockout in MCF7 cells by sequencing and Northern blot. **a** Sanger sequencing was assessed to validate the location and nature of the deletion events. Top and bottom: chromatogram of DNA sequence of the QTRT1 Double Nickase Plasmid working position. Center: the location of the QTRT1-special primers (black) and sgRNA (red and blue) motif are given in different colors. The deletion region is highlighted in gray. **b** Northern blot measurement of Q-modification levels of $tRNA^{His}$ and $tRNA^{Asn}$ using 3-acrylamidophenylboronic acid (APB) gels. Total RNA from WT and QTRT1-KO MFC7 cells (or tumors?) in biological triplicates were separated on denaturing gels containing APB and hybridized with DNA oligo probes complementary to His and Asn tRNAs. Controls are total RNA from HEK293T cells that contain 0% or 100% Q-modified tRNAs (PMID: 29970597).

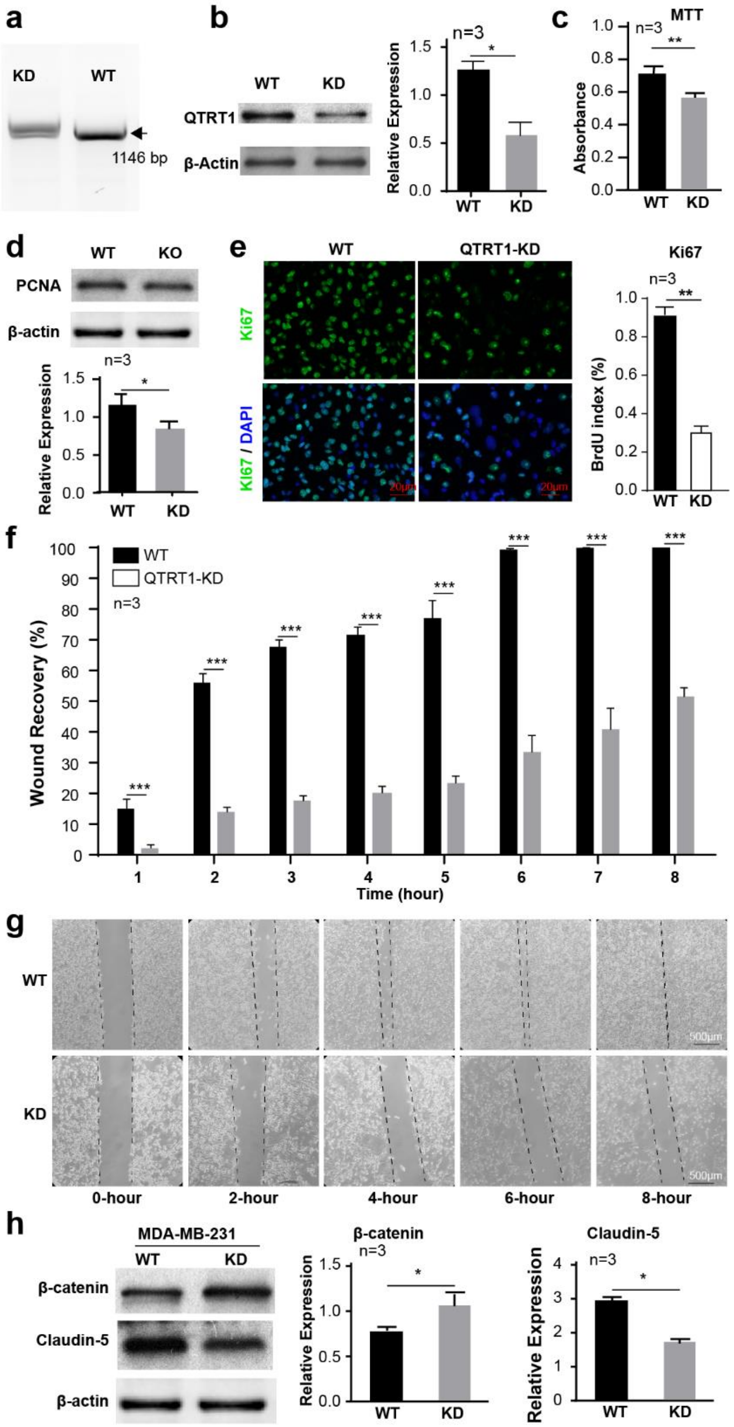

Figure S2. Knockdown of QTRT1 suppressed MDA-MB-231 breast cancer cell proliferation, migration, and altered cell adhesion and tight junctions. **a** The deletion of QTRT1 in MCF7 cells was confirmed using PCR with primers specific for the *QTRT1* gene. **b** Western blot analysis of QTRT1 expression in wildtype (WT), QTRT1-knockdown (KD) MDA-MB-231 cells generated using Double Nickase Plasmids after treating for 72 hours. Mean ± SD, n = 3; * *p*-value < 0.05, two-tailed Welch's *t*-test. **c** MTT assay to show cell proliferation of WT and QTRT1-KD MDA-MB-231 conducted at 48 h after seeding the same number of cells. Mean ± SD, n = 3; ** *p*-value < 0.01, two-tailed Welch's *t*-test. **d**, **e** Cell proliferation markers of PCNA and Ki67 in WT and QTRT1-KD MDA-MB-231 cells were detected using Western blot (**d**) and immunofluorescence staining (**e**), respectively. Immunofluorescence staining of Ki67 (green) and DAPI (blue) were performed in the cells, and Ki67 index (Ki67 stained cells/total cells) was calculated. Mean ± SD, n = 3; ** *p*-value < 0.01, ** *p*-value < 0.01, * *p*-value < 0.05, two-tailed Welch's *t*-test. **f** Wound healing analysis of wildtype and QTRT1-KD MDA-MB-231 cells was shown as the percentage of scratch closure at day-timepoints. Mean ± SD, n =

3; *** p-value < 0.001, two-tailed Welch's t-test. **g** Representative wound healing images were shown. Scale bar is 500 μm. **h** Western analysis of tight junction proteins β-catenin and caludin-5 was performed on QTRT1 knockdown and WT MDA-MB-231 cells. Mean ± SD, n = 3; * p-value < 0.05, two-tailed Welch's t-test.

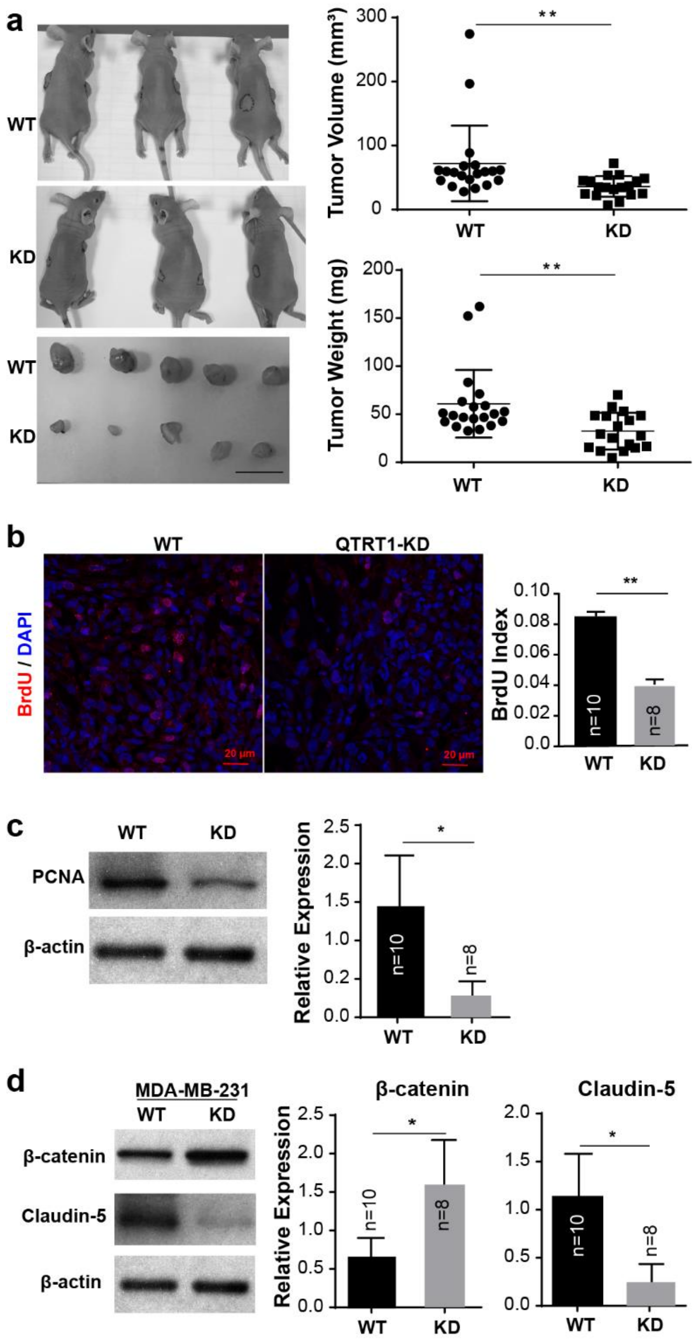

Figure S3. Knockdown of QTRT1 suppressed tumor growth and altered tight junctions in tumors in vivo. **a** The WT and QTRT1-KD MDA-MB-231 cells were injected into nude mice (n = 10), and tumors were harvested 30 days post injection (WT: n = 20; KD: n = 18). The representative photograph of tumors in vivo and in vitro, tumor volume, and tumor weight were shown. The scale bar indicates 10 mm. Each circle represents an individual tumor. Mean ± SD, n = 20 or n = 18; ** p-value < 0.01, two-tailed Welch's t-test. **b** BrdU immunofluorescence staining and index analysis were performed on the tumors from nude mice injected with WT and QTRT1-KD MDA-MB-231 cells. BrdU was detected with sheep monoclonal anti-BrdU and anti-sheep Alexa Fluor 594 (red) antibody. The scale bar is 20 μm. Mean ± SD, n = 10 or n = 8; ** p-value < 0.01, two-tailed Welch's t-test. **c** Western blot analysis of PCNA in tumors harvested from the mice injected with WT and QTRT1-KD MDA-MB-231 cells. The

graph shows the band intensities of PCNA proteins. Mean ± SD n = 10 or n = 8; * p-value < 0.05, two-tailed Welch's t-test. **d** Western blot analysis of tight junction regulators was performed on the tumors isolated from the nude mice model injected with WT and QTRT1-KD MDA-MB-231 breast cancer cells. The graphs show the relative expression of the target proteins detected by Western blot. Mean ± SD, n = 10 or n = 8; * p-value < 0.05, two-tailed Welch's t-test.

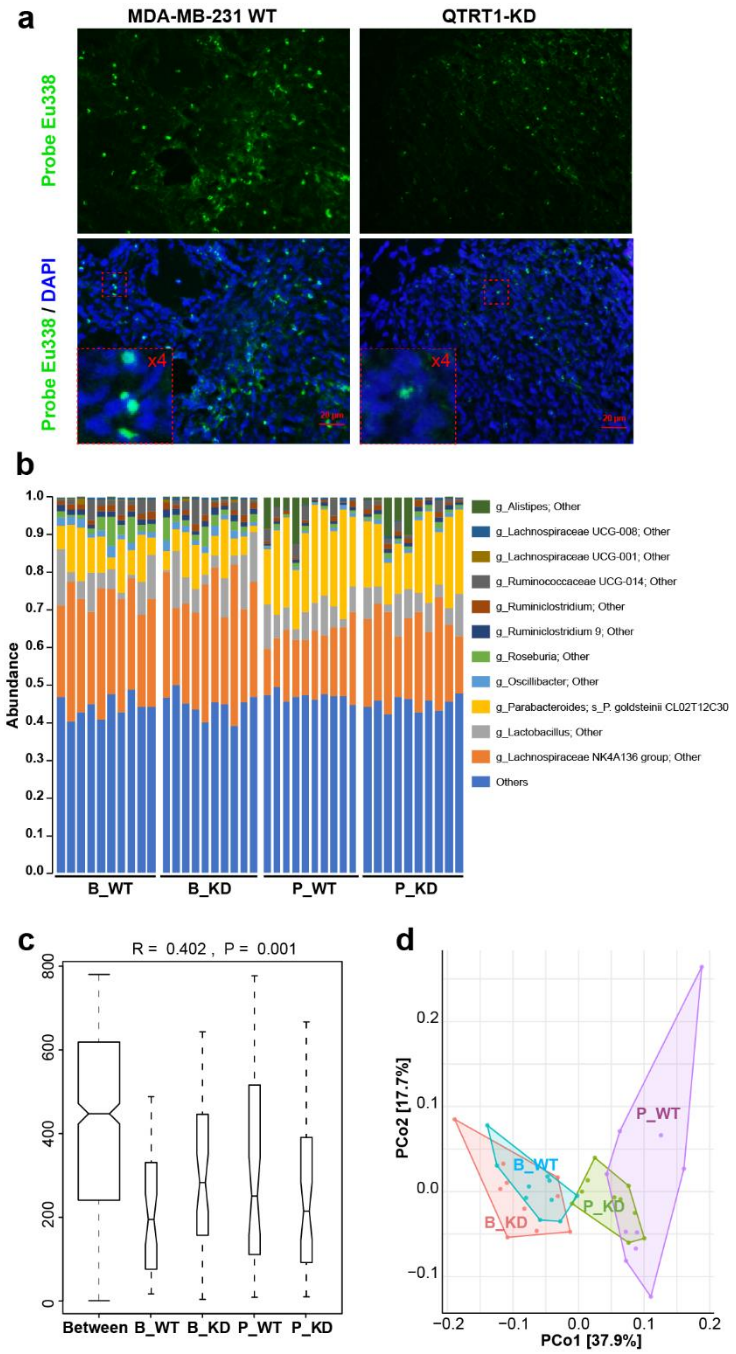

Figure S4. Altered microbiome in tumors and intestines of nude mouse model. **a** Fluorescence in situ hybridization staining with DAPI (blue) and probe EUB338 (green) of the tumors from the nude mice injected with wildtype and QTRT1-KD MDA-MB-231 breast cancer cells. Scale bar is 20 μm. **b** Relative bacterial abundance in species level (s_; the unidentified species were named with super level and other, genus/g_, family/f_) of feces collected from nude mice before (B_WT and B_KD) and post (P_WT and P_KD) injection of wildtype (WT) and QTRT1-KO MDA-MB-231 cells was shown with the top 25 species, and lower ones were grouped as "others". Species were colored using the key as list in the right side. Each bar represents individual mice (n = 10 each group). **c** Plots of between and within means of Sorensen dissimilarity. The analysis of similarity (ANOSIM) was performed on

between (Between) and within groups of before QTRT1-KD MDA-MB-231 cell injection (B_KD), before WT cell injection (B_WT), post QTRT1-KD cell injection (P_KD), and post WT cell injection (P_WT). n = 10 per group. **d** The principal coordinates analysis (PCoA) plot of the mouse feces was produced to inspect the homogeneity of multivariate dispersions. The samples collected before (B_WT and B_KO) and post (P_WT and P_KD) MDA-MB-231 cell injection were colored in the illustration. n = 10 per group.

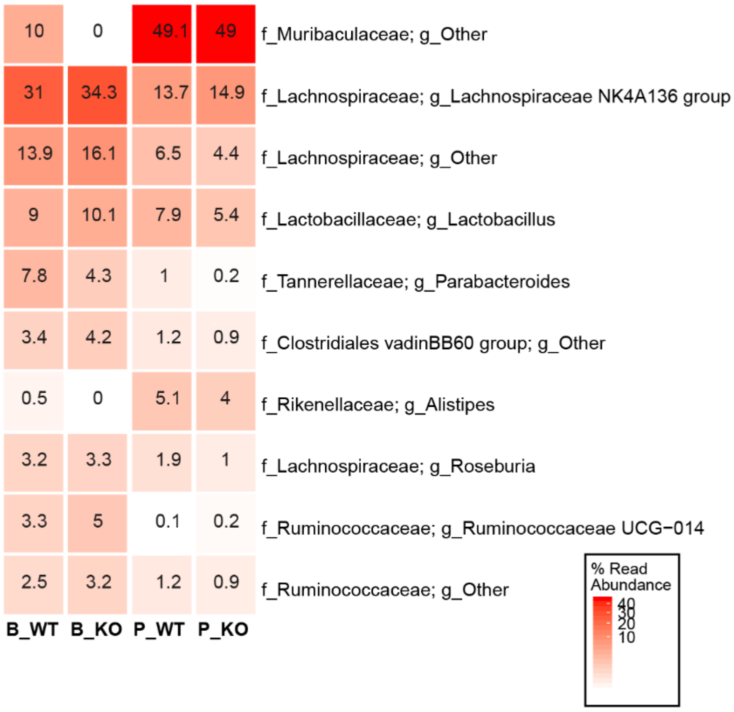

Figure S5. Heatmap of the bacterial percentage abundance in the intestine of nude mice. The representative microbial taxa found in the fecal communities of mouse model before (B_WT and B_KO) and post (P_WT and P_KO) cell injection of wildtype (WT) and QTRT1-KO (KO) MCF7 cells were illustrated in the heatmap. Both taxonomic family (f_) and genus (g_) level were assessed to identify the bacteria. The percentile read abundance was illustrated in color as the color bar in the right and together with the number inside the blocks.

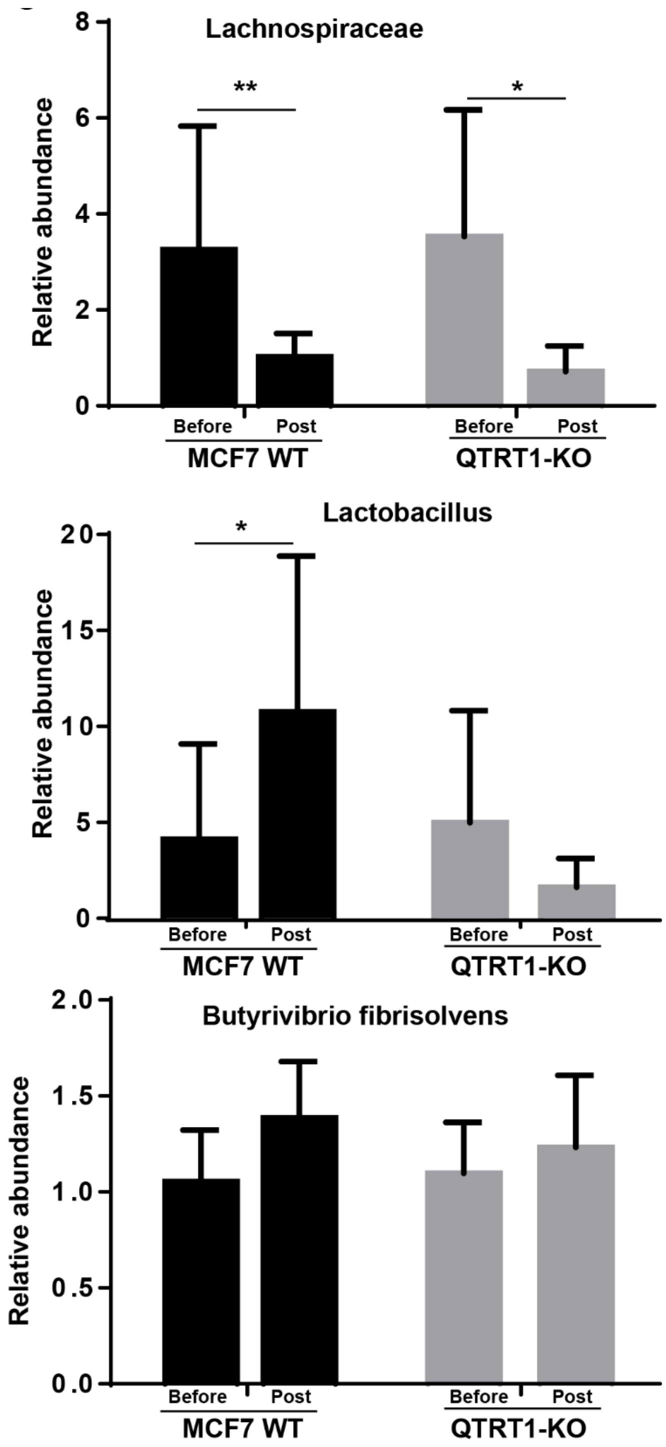

Figure S6. Relative abundance of intestinal bacteria detected with real-time PCR. Using the real-time PCR with specific primers, the relative abundance of Lachnospiraceae, *Lactobacillus*, and *Butyrivibrio fibrisolvens* was evaluated with the feces samples. Mean ± SD, n = 10; * p-value < 0.05, ** p-value < 0.01, two-tailed Welch's t-test.
