# Supplementary material for: tRNA Queuosine Modification Enzyme Modulates the Growth and Microbiome Recruitment to Breast Tumors
Source: Cancers (Basel). 2020 Mar 9;12(3):628. doi: 10.3390/cancers12030628 (PMC7139606; doi:10.3390/cancers12030628)

1. QTRT1-knockout (KO) in MCF7 Cells

1.1 Cell culture

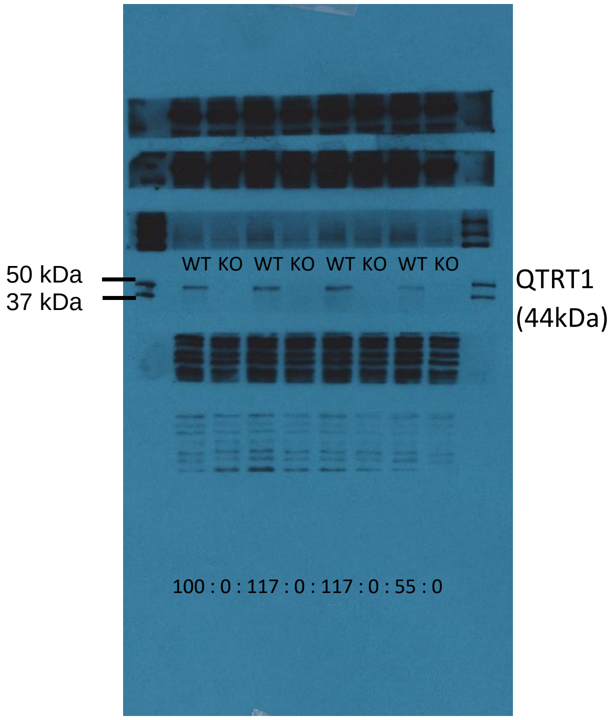

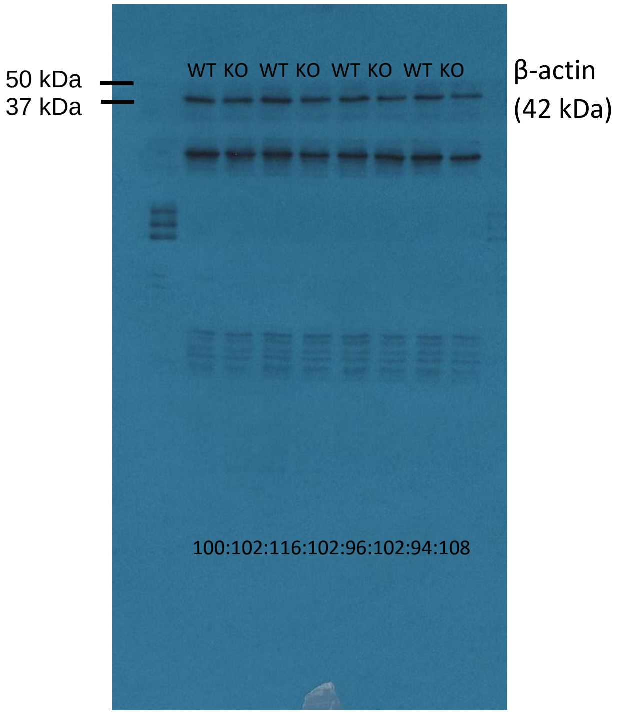

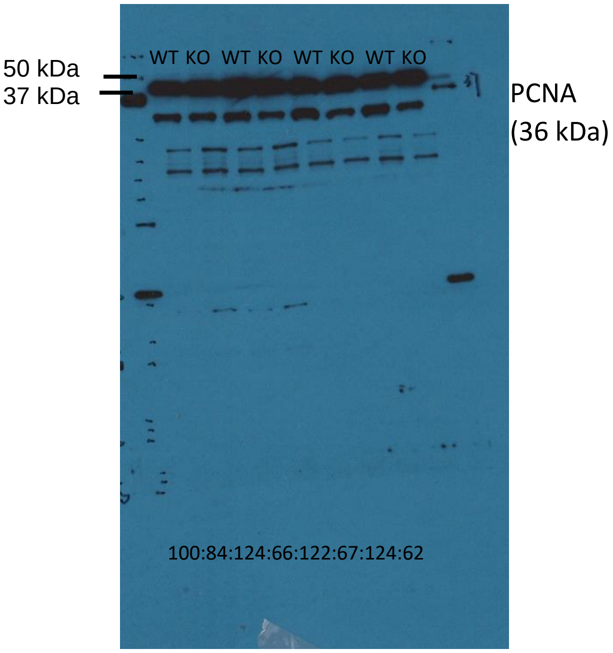

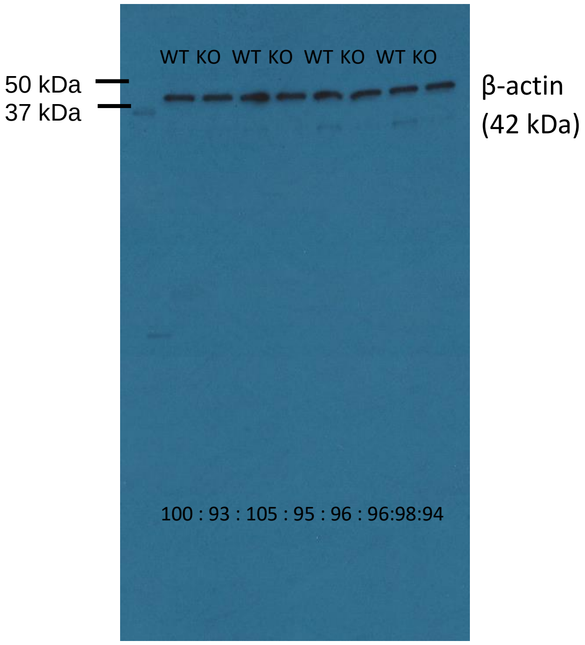

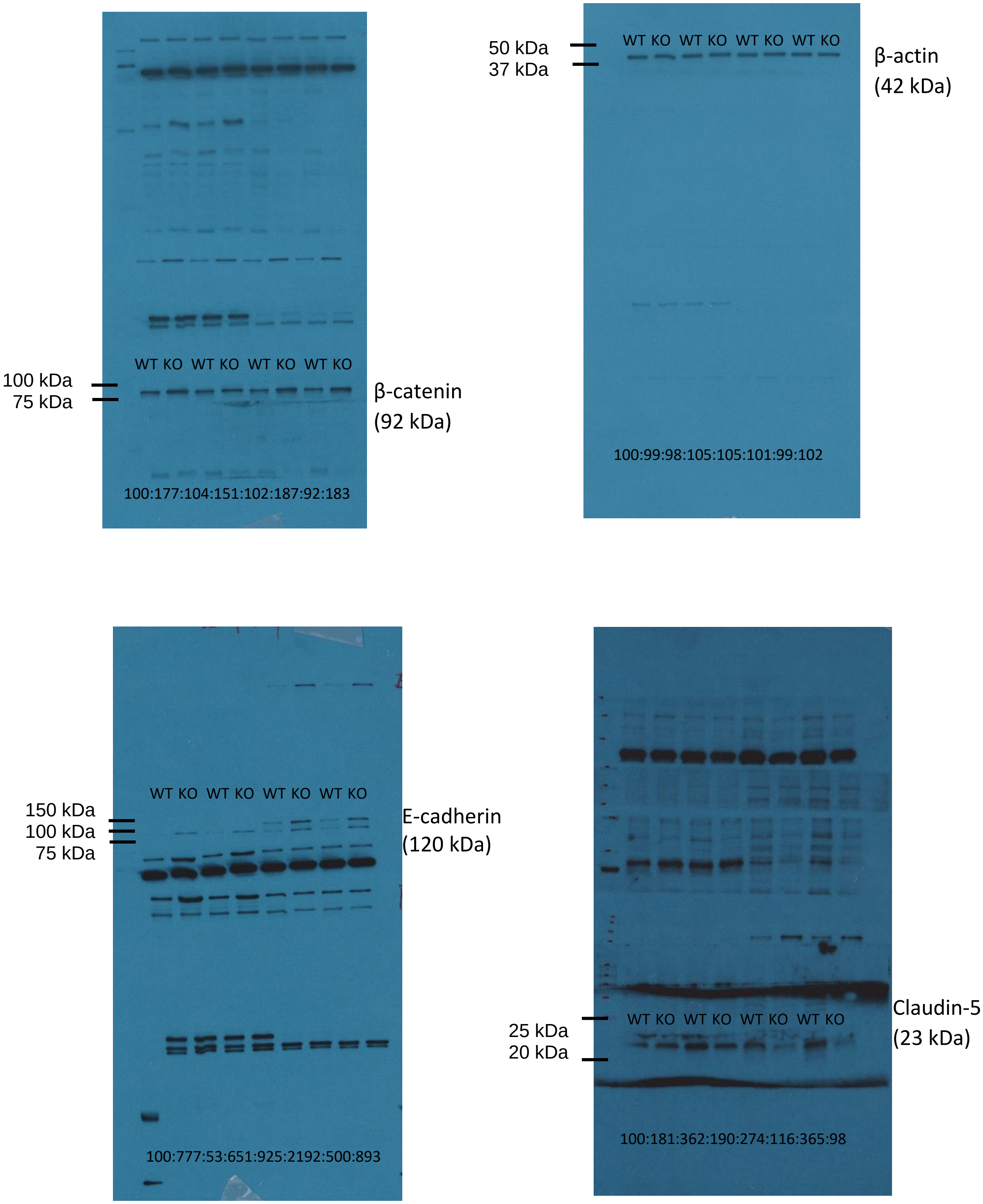

WT KO WT KO WT KO WT KO
100 kDa
75 kDa
β-catenin
(92 kDa)
100:177:104:151:102:187:92:183
50 kDa
37 kDa
WT KO WT KO WT KO WT KO
β-actin
(42 kDa)
100:99:98:105:105:101:99:102
WT KO WT KO WT KO WT KO
150 kDa
100 kDa
75 kDa
E-cadherin
(120 kDa)
100:777:53:651:925:2192:500:893
25 kDa
20 kDa
WT KO WT KO WT KO WT KO
Claudin-5
(23 kDa)
100:181:362:190:274:116:365:98

1.2 Nude mouse model

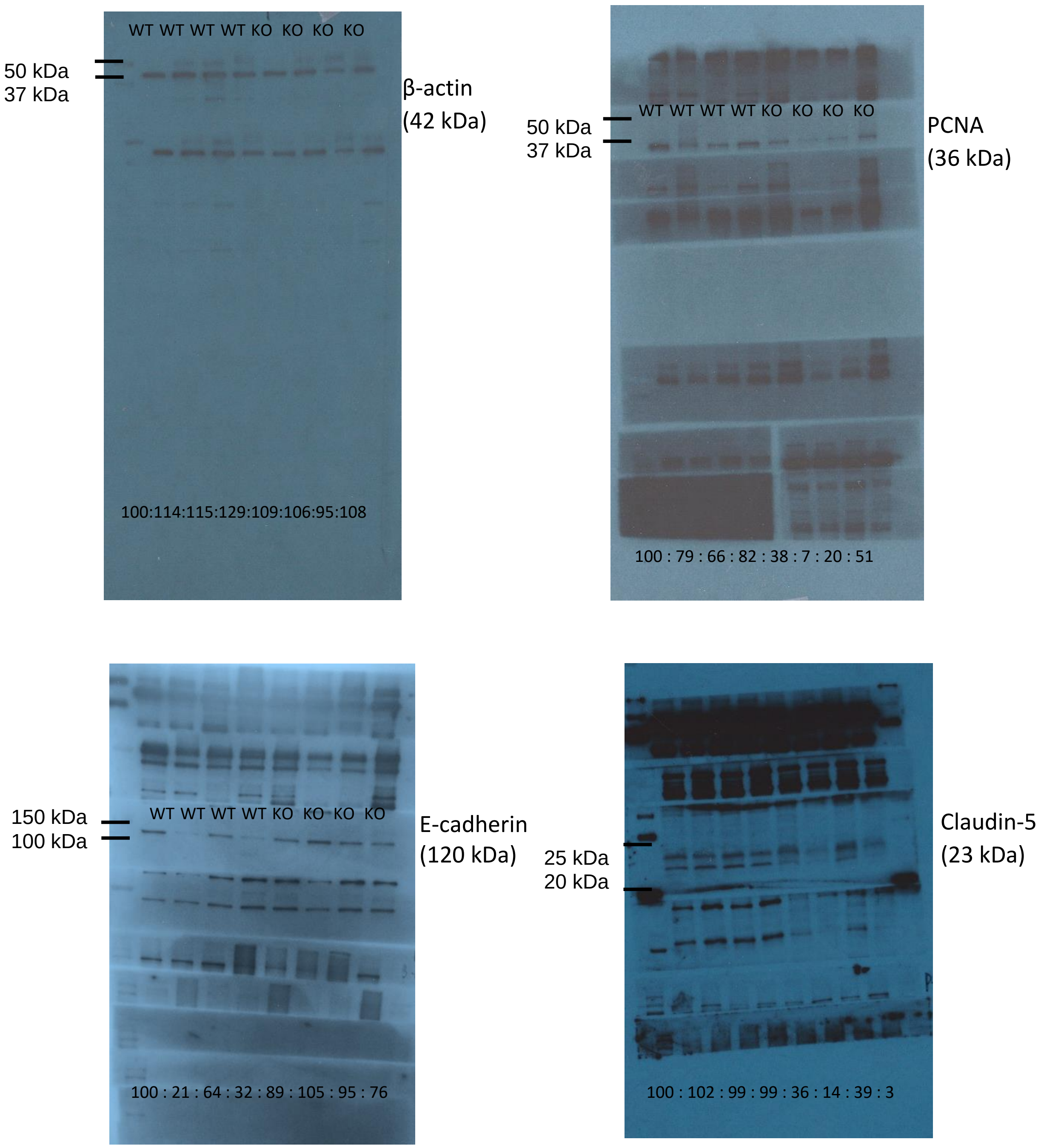

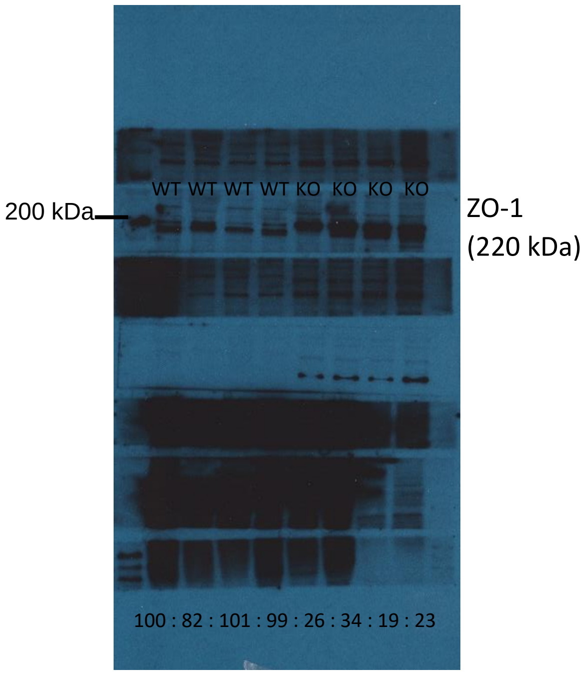
WT WT WT WT KO KO KO KO
200 kDa
ZO-1
(220 kDa)
100 : 82 : 101 : 99 : 26 : 34 : 19 : 23

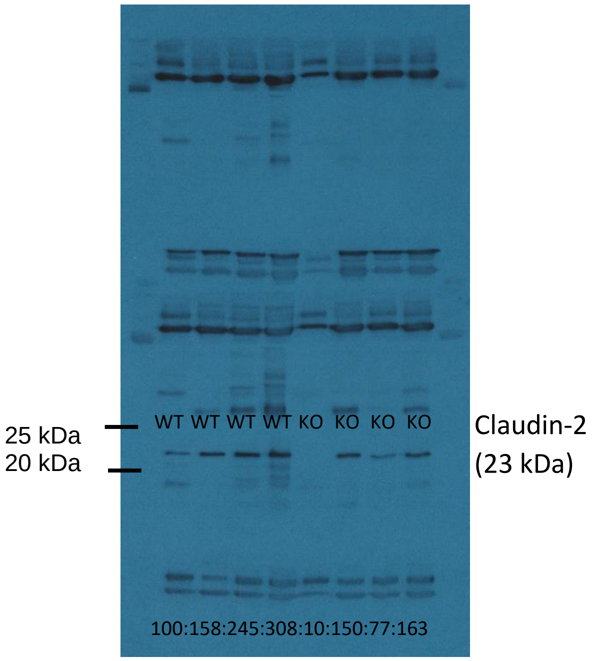
WT WT WT WT KO KO KO KO
25 kDa
20 kDa
Claudin-2
(23 kDa)
100:158:245:308:10:150:77:163

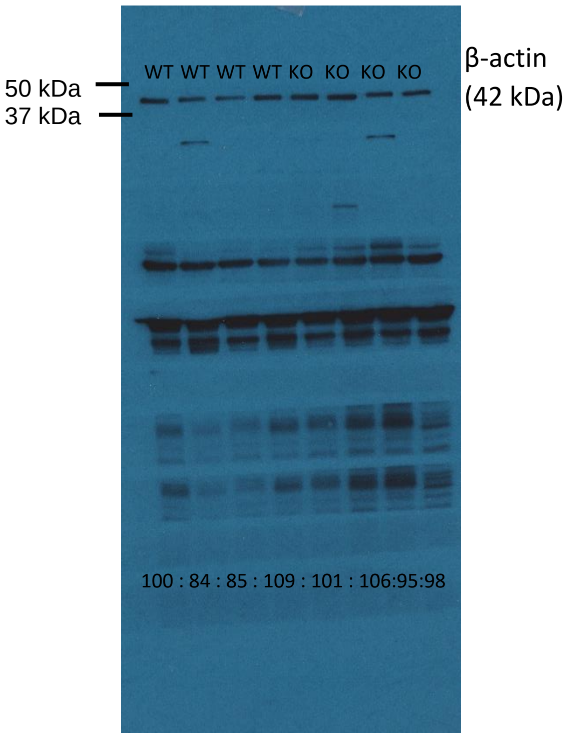
WT WT WT WT KO KO KO KO
50 kDa
37 kDa
β-actin
(42 kDa)
100 : 84 : 85 : 109 : 101 : 106:95:98

2. QTRT1-knockdown (KD) in MDA-MB-231 Cells

2.1 Cell culture

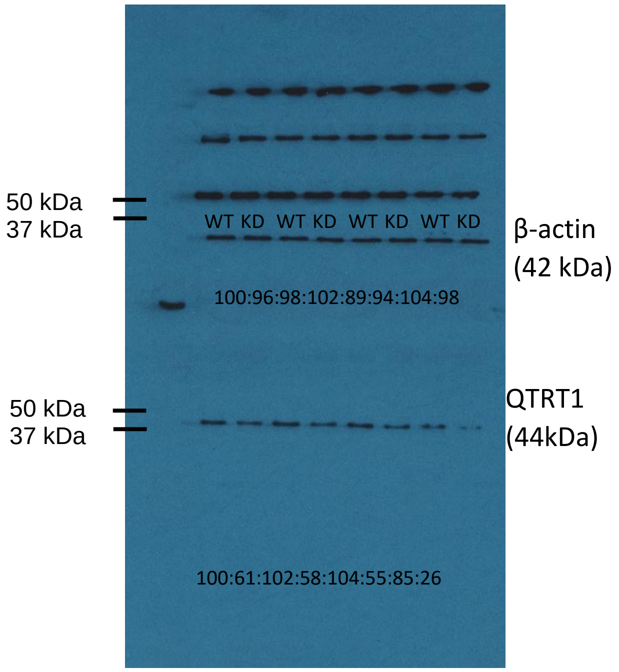

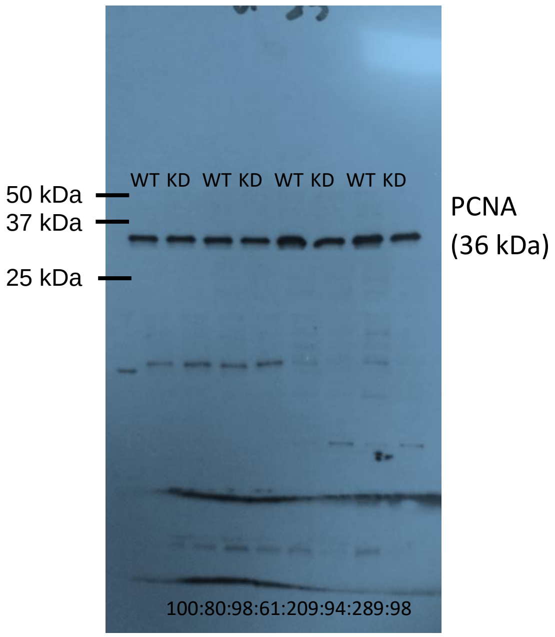

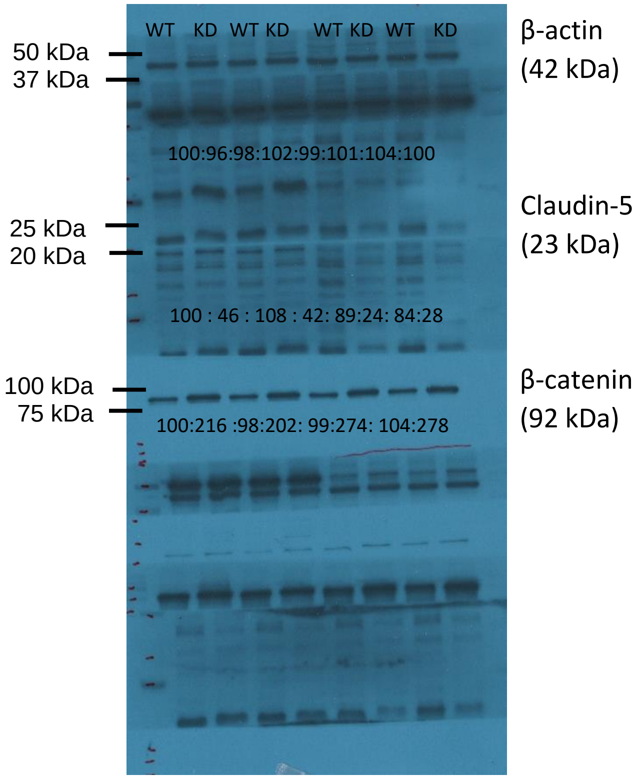

2.2 Nude mouse model

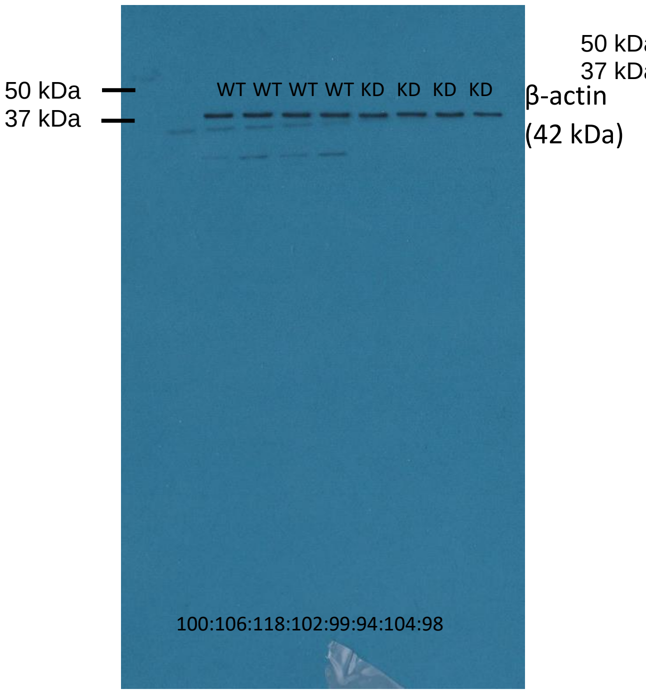

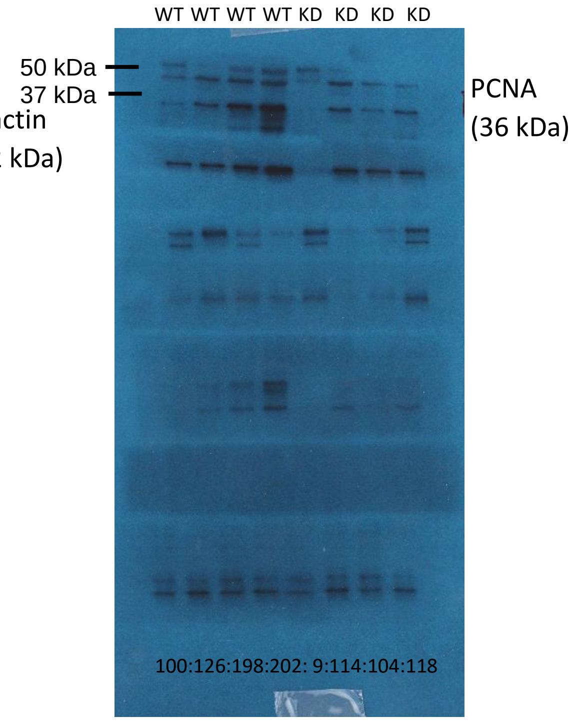

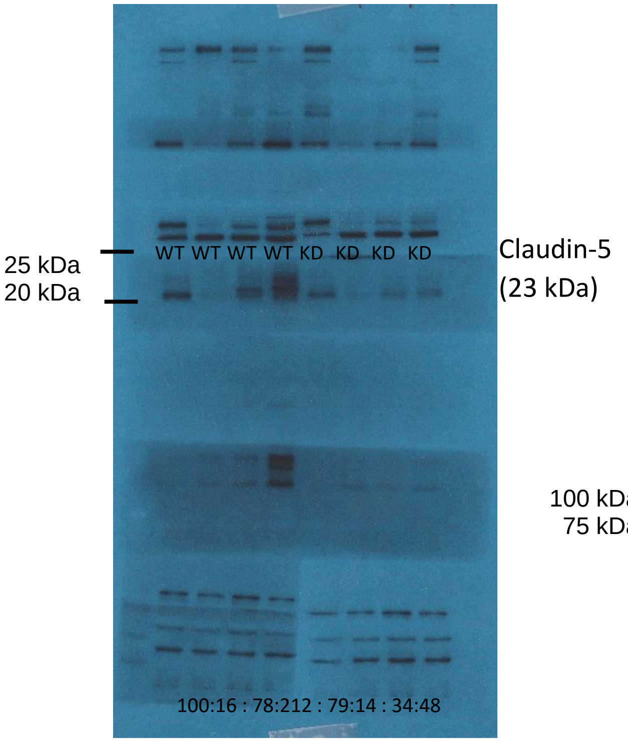

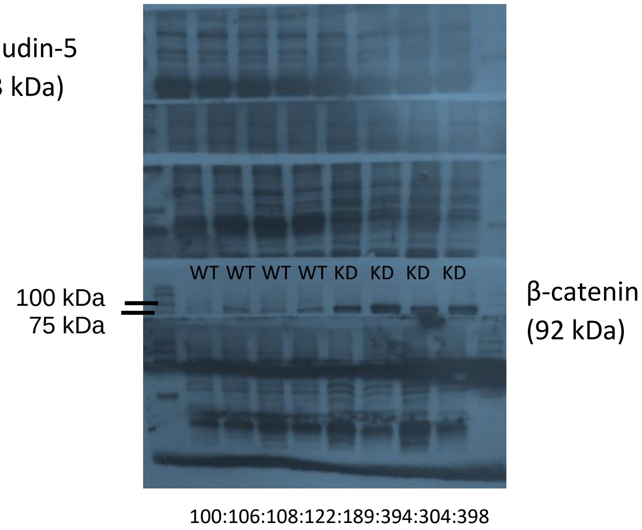

Supplement: Supplementary file 1 [file cancers-12-00628-s001.zip › Western blot source data.pdf]
